# Construction of Multiple Asymmetric Catalytic Sites on Carbon Nitrides Toward Efficient Solar Hydrogen Peroxide Production

**DOI:** 10.1002/advs.202513453

**Published:** 2025-09-16

**Authors:** Siyu Sun, Feng Gao, Hu Yang

**Affiliations:** ^1^ State Key Laboratory of Water Pollution Control and Green Resource Recycling School of the Environment Nanjing University Nanjing 210023 P. R. China; ^2^ Department of Materials Science and Engineering Jiangsu Key Laboratory of Artificial Functional Materials Collaborative Innovation Center of Advanced Microstructures College of Engineering and Applied Sciences Nanjing University Nanjing 210023 P. R. China

**Keywords:** asymmetric coordination, H_2_O_2_ photosynthesis, multiple active sites, O_2_ reduction, single‐atom photocatalysis

## Abstract

Solar‐driven hydrogen peroxide (H_2_O_2_) production represents a sustainable alternative to energy‐intensive industrial processes, yet its efficiency is hindered by poor charge separation and sluggish reaction kinetics. Here, a structurally adaptive strategy is proposed to create highly asymmetric multi‐active‐site architectures by synergistically integrating sulfur (S) dopants and single‐atom zinc (Zn) species into the repeating units of 1D and 2D carbon nitride (C_3_N_4_) frameworks, i.e., C_3_N_4_ nanotube (CNT) and sheet (CNS). In this structure, S/Zn and N/O atoms contribute to the conduction and valence bands, respectively, providing multiple charge transfer pathways for photogenerated carriers to achieve efficient spatial separation. The electron delocalization promoted by the highly asymmetric configuration optimizes O_2_ adsorption on Zn atoms and reduces the energy barrier for ^*^OOH intermediate formation. Consequently, the optimized S‐CNS‐Zn and S‐CNT‐Zn catalysts exhibit remarkable H_2_O_2_ evolution rates of 1724 and 2708 µmol g^−1^ h^−1^, ≈72.1 and 17.5 fold higher than pristine C_3_N_4_, with an apparent quantum yield of 6.28% and 9.88% at 420 nm and solar‐to‐chemical conversion efficiency of 0.37% and 0.52%, respectively, surpassing most previously reported values. This work provides atomic insights for the design of multiple asymmetric catalytic sites.

## Graphical abstract

A structurally adaptive strategy has been developed to construct multiple asymmetric catalytic sites (S, Zn, N, O) within 1D and 2D carbon nitride. The asymmetric configuration promotes spatial separation of charge carriers and enhances O_2_ adsorption and ^*^OOH formation through improved electron delocalization, significantly boosting photocatalytic H_2_O_2_ production under visible light irradiation.

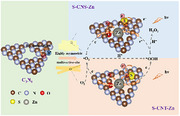

## 1 Introduction

Hydrogen peroxide (H_2_O_2_), a versatile green oxidant, plays a pivotal role in environmental remediation, chemical synthesis, and energy conversion.^[^ ^1^ ^]^ However, the conventional anthraquinone process for industrial H_2_O_2_ production suffers from high energy consumption, hazardous by‐products, and complex infrastructure, severely limiting its sustainability.^[^ ^2^, ^3^, ^4^ ^]^ Photocatalytic H_2_O_2_ synthesis via the solar‐driven oxygen reduction reaction (ORR) has emerged as a promising alternative, offering a renewable and eco‐friendly route to harness solar energy for chemical production.^[^ ^5^ ^,^ ^6^ ^]^

Among photocatalysts, polymeric carbon nitride (C_3_N_4_) has garnered considerable attention as a metal‐free photocatalyst due to its tunable bandgap, chemical stability, and earth‐abundant composition.^[^ ^7^, ^8^ ^]^ However, pristine C_3_N_4_ typically exhibits a highly symmetric structure, with carbon (C) and nitrogen (N) atoms arranged periodically within its triazine framework.^[^ ^9^ ^]^ The uniform distribution of localized electrons suggests a difficult electron transfer within the layers due to the spatial proximity of oxidation and reduction sites.^[^ ^10^ ^]^ These inherent limitations lead to poor charge separation efficiency and weak O_2_ adsorption capacity, significantly hindering the practical application of C_3_N_4_ in H_2_O_2_ production.^[^ ^11^, ^12^ ^]^ Recent efforts to enhance its photocatalytic performance have focused on heteroatom doping (e.g., B, S, and P)^[^ ^13^, ^14^, ^15^, ^16^ ^]^ and single‐atom engineering,^[^ ^17^, ^18^, ^19^ ^]^ which modulate electronic properties and disrupt the symmetric structure of C_3_N_4_.^[^ ^20^, ^21^ ^]^ Although some materials doped with heteroatoms or modified with single metal atoms exhibit asymmetric structural configurations, their localized mono‐ or dual‐site asymmetry often leads to limited electron delocalization. This typically causes sluggish separation and rapid recombination of photogenerated carriers, thereby weakening photoreduction reactions.^[^ ^22^, ^23^, ^24^ ^]^ For example, single S‐doped C_3_N_4_ relies on a single pathway for carrier transport during H_2_O_2_ synthesis, the performance of which was improved by only 1.30 times.^[^ ^13^ ^]^ In the reported Zn‐N_4_ system, Zn single atoms show a symmetric coordination configuration in the catalyst, but their limited electron delocalization causes a slow carrier separation, resulting in a mere 2.32 fold improvement in H_2_O_2_ synthesis performance.^[^ ^25^ ^]^ By contrast, the introduction of multiple asymmetric sites within triazine repeating units can generate a highly asymmetric electronic structure, extend electron delocalization, and promote spatial separation of charge carriers in C_3_N_4_, thereby improving photocatalytic performance.^[^ ^24^ ^]^ Nevertheless, precise control over atomic configurations to synergistically integrate multiple modification sites for constructing highly asymmetric electronic structures remains a major challenge.

Herein, a structurally adaptive two‐step strategy was developed to construct multiple asymmetric catalytic sites on the triazine repeating units of CNS and CNT architectures through sulfur (S) doping and zinc (Zn) single‐atom anchoring (denoted as S‐CNS‐Zn and S‐CNT‐Zn, respectively). Advanced characterization techniques have shown that in S‐CNS‐Zn and S‐CNT‐Zn, S substitutes the N_2C_ and N_3C_ sites, and the Zn single atoms exist in the form of Zn–N_3_O. Through a series of experiments and theoretical calculations, the charge transfer pathways and photocatalytic mechanisms for H_2_O_2_ production have been thoroughly investigated. The asymmetric N/O/S/Zn active sites endow the catalysts with higher intrinsic activity and alter the electronic structure of C_3_N_4_, achieving multi‐path separation of charge carriers and facilitating O_2_ adsorption as well as the subsequent formation of the key intermediate ∗OOH. By bridging the gap between atomic‐level design and macroscopic performance, this study not only offers valuable guidance for the precise design of high‐efficiency C_3_N_4_‐based photocatalysts for H_2_O_2_ production in the future but also provides fundamental physical insights into the atomic‐scale mechanisms of multiple asymmetric active sites.

## 2 Results and Discussion

### 2.1 Synthesis and Structural Characterization

**Figure 1:**
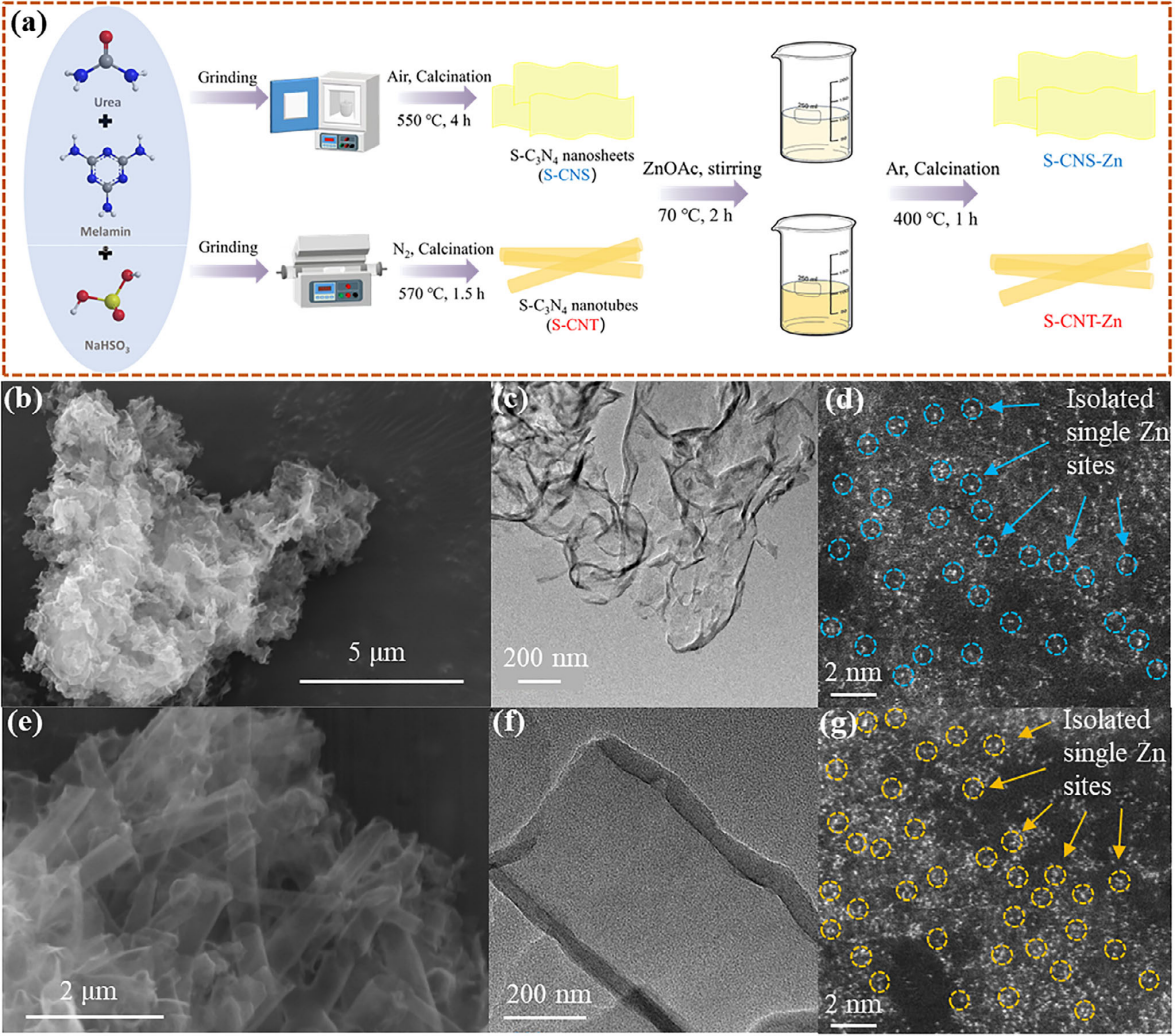
a) Schematic illustration of the synthesis route for S‐CNS, S‐CNT, S‐CNS‐Zn, and S‐CNT‐Zn, b,e) SEM, c,f) TEM, and d,g) aberration‐corrected HAADF‐STEM images of (b,c,d) S‐CNS‐Zn and (e,f,g) S‐CNT‐Zn, respectively.

S‐CNS‐Zn and S‐CNT‐Zn exhibiting mesoscopic asymmetry were prepared via a two‐step approach. Detailed experimental procedures are provided in the Supporting Information. According to previous reports, adjusting the ambient atmosphere can regulate the formation of C_3_N_4_ with different morphologies.^[^ ^26^, ^27^ ^]^ As illustrated in **Figure** **1**a, a series of sulfur‐doped flake‐like and tubular C_3_N_4_ materials with S‐catalytic sites (S‐CNS‐x and S‐CNT‐x, x = 1, 2, 3, 4) were first synthesized by adjusting the annealing atmosphere and the stoichiometric ratio of precursors.^[^ ^13^, ^15^, ^28^, ^29^ ^]^ Among them, S‐CNS‐2 and S‐CNT‐2 (hereinafter referred to as S‐CNS and S‐CNT for short) were the optimal ones in each series and exhibited the highest H_2_O_2_ generation activity (Figure S1a,b, Supporting Information), with the yields of 36.4% and 27.0% respectively. In addition, the X‐ray diffraction (XRD) patterns (Figure S2a,b, Supporting Information) indicate that different stoichiometric ratios of the S atom do not alter the original sheet‐like or tubular crystal forms under the same ambient atmosphere. These materials were subsequently mixed with zinc acetate dihydrate (Zn(OAc)_2_) in an ethanol solution at 70 °C and annealed at 400 °C under an argon atmosphere.^[^ ^30^, ^31^ ^]^ Through this process, zinc species were likely immobilized onto S‐CNS and S‐CNT, forming single‐atom sites while introducing oxygen coordination near the Zn centers,^[^ ^25^ ^]^ thereby creating samples with multiple asymmetric catalytic sites. Among the resultant products, S‐CNS‐Zn‐2 and S‐CNT‐Zn‐2 (hereinafter referred to as S‐CNS‐Zn and S‐CNT‐Zn for short) showed the highest H_2_O_2_ generation activity (Figure S1c,d, Supporting Information), with yields of 92.0% and 85.6% respectively. Similarly, as demonstrated in Figure S2c,d (Supporting Information), different stoichiometric ratios of Zn(OAc)_2_ do not alter the original lamellar or tubular morphologies of C_3_N_4_; however, as the fed amount of Zn(OAc)_2_ increases further, the distinct XRD characteristic peaks corresponding to ZnO appeared at 31.8°, 34.0°, and 36.3° (Figure S2c,d, Supporting Information) indicate that excess Zn(OAc)_2_ leads to the formation of ZnO.^[^ ^32^ ^]^ Thus, the amount of Zn(OAc)_2_ should be controlled well to avoid the formation of ZnO in the preparation of the Zn‐loaded C_3_N_4_ materials. The Brunauer–Emmett–Teller (BET) surface area of S‐CNS‐Zn and S‐CNT‐Zn was measured to be 64 and 31 m^2^/g, respectively (Figure S3, Supporting Information). The higher specific surface area of S‐CNS‐Zn may be attributed to two facts: 1) the inherent advantage of the 2D sheet‐like structure in providing a high exposed surface area, but 2) the masking of the outer surface caused by the tight stacking between the tubes of the 1D tubular structure.^[^ ^27^ ^]^ Additionally, pristine flake‐like and tubular C_3_N_4_ (CNS and CNT) were prepared via thermal polymerization for comparison.^[^ ^33^, ^34^ ^]^

Comprehensive characterizations were employed to confirm the successful fabrication of varied 1D and 2D C_3_N_4_‐based materials while maintaining their structural integrity. Scanning electron microscopy (SEM) and transmission electron microscopy (TEM) images revealed that the sulfur‐doped modifications and subsequent zinc loading retained the initial flake‐like and tubular morphologies of C_3_N_4_ (Figure 1b,c,e,f; Figures S4–S7, Supporting Information), with the diameter of the nanotubes ranging from 100 to 400 nm. In comparison to S‐CNS(T)‐Zn‐4 with overdosed Zn(OAc)_2_ in the preparation process (Figure S8, Supporting Information), it can be preliminarily determined that there are no metal nanoparticles on the surface of S‐CNS(T)‐Zn, based on their SEM and TEM images. In their XRD patterns (Figure S9, Supporting Information), no obvious signals corresponding to zinc‐based agglomerates were detected, revealing the absence of detectable Zn nanoparticles or clusters, and Zn may thus exist in the form of single atoms in the catalyst.^[^ ^35^ ^]^ Fourier‐transform infrared (FTIR) spectroscopy revealed a new peak at 707.98 cm^−1^ (attributed to C─S stretching) after sulfur incorporation, and spectral consistency was maintained before and after zinc loading (Figure S10, Supporting Information).^[^ ^36^ ^]^ The C─S signal in S‐CNS was absent, possibly because its sulfur content was below detection limits. Atomic‐scale visualization of zinc species was achieved through aberration‐corrected high‐angle annular dark‐field scanning transmission electron microscopy (AC‐HAADF‐STEM). The presence of uniformly dispersed bright spots in the images confirmed the single‐atom dispersion of zinc sites within the S‐CNS‐Zn and S‐CNT‐Zn frameworks (Figure 1d,g). Energy‐dispersive X‐ray spectroscopy further demonstrated the homogeneous distributions of C, N, S, Zn, and O throughout the tubular and flake‐like architectures (Figure S11, Supporting Information), providing additional evidence for the uniform dispersion of zinc sites on the substrate.

**Figure 2:**
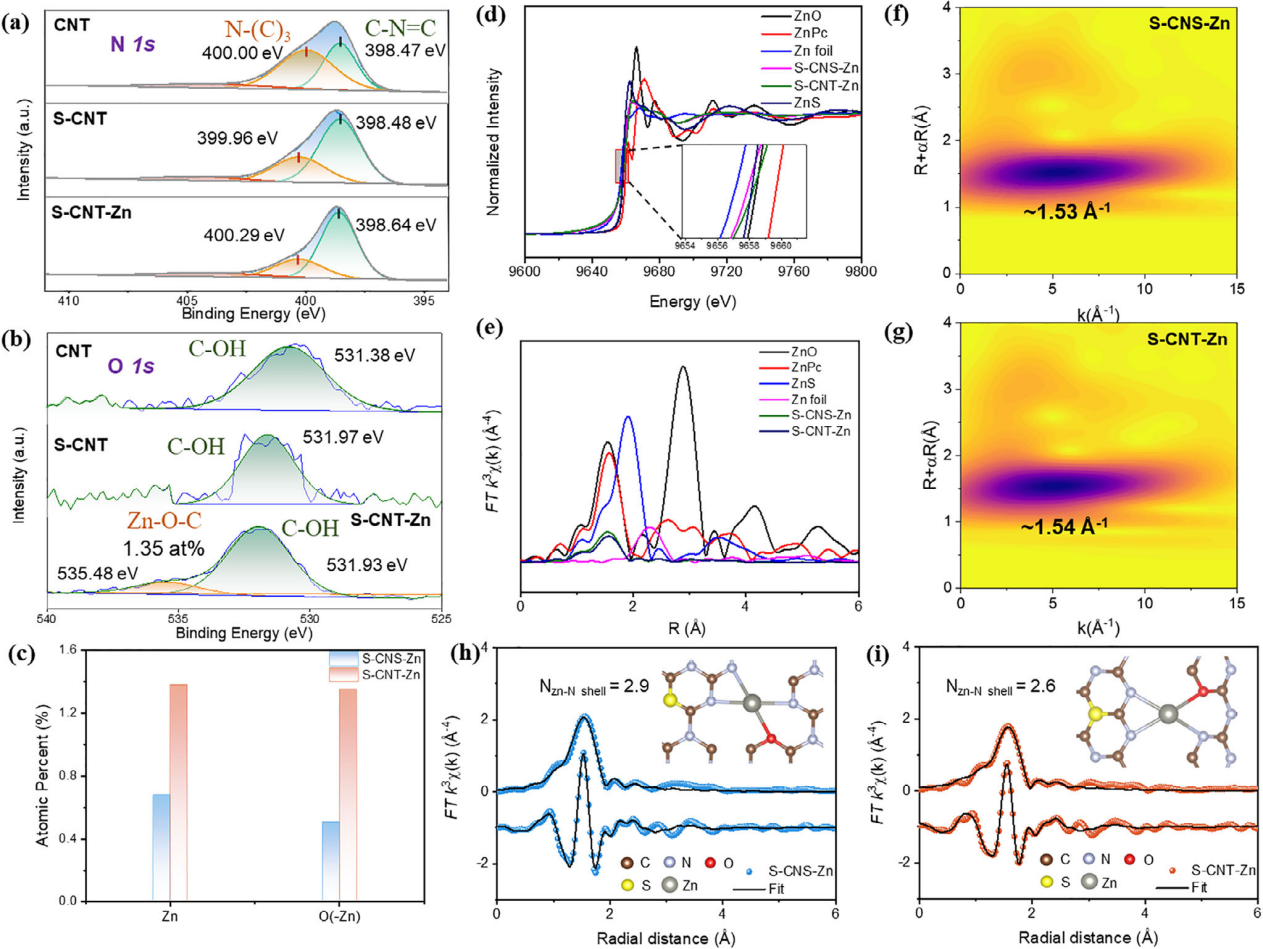
a,b) High‐resolution N 1s and O1s XPS spectra of CNT, S‐CNT, and S‐CNT‐Zn. c) Atomic percents of Zn and O (in the form of Zn─O─C) in S‐CNS‐Zn and S‐CNT‐Zn obtained from XPS results. d) Zn K‐edge XANES spectra of as‐prepared samples and references. The inset shows the marked part of Figure 2d. e) Fourier transform curves of Zn K‐edge EXAFS spectra of as‐prepared samples and references. Wavelet transforms of the EXAFS signals of f) S‐CNS‐Zn and g) S‐CNT‐Zn. The Zn K‐edge EXAFS fitting results of h) S‐CNS‐Zn and i) S‐CNT‐Zn, respectively.

X‐ray photoelectron spectroscopy (XPS) survey analysis roughly determined the elemental composition on the surface, revealing sulfur (0.83at%), oxygen (8.79at%), and zinc (1.38at%) incorporation in S‐CNT‐Zn (Figure S12, Supporting Information). Notably, the C 1s XPS spectrum exhibited a slight positive binding energy shift upon sulfur doping in CNS and CNT, which could be attributed to the decreased electron density of carbon species resulting from C─S bond formation (Figure S13, Supporting Information).^[^ ^37^, ^38^ ^]^ This observation was in agreement with the appearance of a characteristic C─S stretching vibration in their FTIR spectra (Figure S10, Supporting Information). The combined spectroscopic evidence confirmed that sulfur incorporation primarily occurred through covalent bonding with carbon, while maintaining the structural integrity of the host material. Remarkably, upon Zn single‐atom loading on S‐CNS and S‐CNT, the high‐resolution N 1s XPS spectra (**Figure** **2**a; Figure S14a, Supporting Information) revealed significant positive shifts in binding energies for C─N_3_ and C─N═C nitrogen species. This result suggested these nitrogen moieties served as primary coordination sites for stabilizing the Zn single atoms.^[^ ^39^, ^40^ ^]^ Besides, the N 1s XPS spectra revealed material‐dependent nitrogen speciation changes: sulfur doping in S‐CNT preferentially substituted N_3C_ sites (increasing N_2C_/N_3C_ ratio, Table S1, Supporting Information), whereas S‐CNS showed the opposite N_2C_ substitution behavior.^[^ ^37^ ^]^ Notably, an additional oxygen species emerged at 535.5 and 535.4 eV in S‐CNS‐Zn and S‐CNT‐Zn, respectively (Figure 2b; Figure S14b, Supporting Information), beyond the characteristic C─OH groups observed in their pristine and sulfur‐doped C_3_N_4_ materials. This distinct spectral feature was conclusively assigned to Zn─O─C species formed through the anchoring of acetate‐derived oxygen (from Zn(OAc)_2_) onto C_3_N_4_.^[^ ^41^, ^42^ ^]^ Notably, the atomic percentages of oxygen in the Zn─O─C configuration closely matched those of the Zn species (ratios of 1.33:1 and 1.02:1, Figure 2c), providing strong evidence for monodentate oxygen coordination. Thus, each Zn site is bonded to approximately one oxygen atom. Collectively, our spectroscopic data demonstrated that isolated Zn sites in S‐CNS‐Zn and S‐CNT‐Zn adopted a unique mixed coordination environment, involving multiple nitrogen donors (as evidenced by N 1s XPS shifts) and a single oxygen ligand (via Zn─O─C bonding). The coexistence of these coordination modes created an asymmetric electronic structure that may significantly influence the material's catalytic properties.

The chemical composition, oxidation states, and atomic configuration of S‐CNS‐Zn and S‐CNT‐Zn were comprehensively characterized using X‐ray absorption fine structure (XAFS) spectroscopy.^[^ ^43^ ^]^ The Zn K‐edge X‐ray absorption near‐edge structure (XANES) spectra (Figure 2d) demonstrated nearly identical absorption edge energies for S‐CNS‐Zn and S‐CNT‐Zn, and this finding was further corroborated by high‐resolution Zn 2p XPS spectra (Figure S15, Supporting Information). Their edge positions closely resembled that of ZnO (Figure 2d), indicating a predominant +2 oxidation state for Zn species in both materials.^[^ ^19^ ^]^ Extended XAFS (EXAFS) spectroscopy provided detailed insights into the local coordination environment of isolated Zn sites in S‐CNT‐Zn and S‐CNS‐Zn. As shown in Figure 2e, the Fourier‐transformed EXAFS spectra of the prepared samples exhibited a dominant peak at ≈1.56 Å, corresponding to either Zn─N coordination (similar to zinc phthalocyanine, ZnPc) or Zn─O bonding (comparable to ZnO reference).^[^ ^18^, ^44^, ^45^ ^]^ Notably, no detectable signal was observed at 2.32Å (characteristic of Zn─Zn metallic bonds), which excluded the formation of zinc nanoparticles or the presence of zinc clusters. The similar EXAFS profiles between S‐CNT‐Zn and S‐CNS‐Zn suggested comparable coordination environments despite their different C_3_N_4_ matrices. Through wavelet transform (WT) EXAFS analysis, we successfully elucidated the precise coordination structure of Zn sites in S‐CNS‐Zn and S‐CNT‐Zn. The WT contour plots (Figure 2f,g) revealed characteristic features at radial distances of 1.53–1.54 Å that closely resembled the signature Zn─N_4_ coordination observed in zinc phthalocyanine (ZnPc, 1.56 Å^−1^), while showing distinct differences from Zn foil references (Figure S16, Supporting Information).^[^ ^17^, ^46^ ^]^ Thus, S‐CNS‐Zn and S‐CNT‐Zn possessed a similar Zn configuration to ZnPc (Zn─N_4_). Quantitative EXAFS fitting further demonstrated that Zn sites in S‐CNS‐Zn and S‐CNT‐Zn adopted a unique mixed coordination environment, bonding with ≈3 nitrogen atoms (coordination number = 2.9 or 2.6) and 1 oxygen atom (coordination number = 1.0), i.e., Zn─N_3_O, (Figure 2h,i; Table S2, Supporting Information), which was fully consistent with the nearly identical atomic percentages observed for Zn and oxygen in Zn─O─C configuration (Figure 2c).^[^ ^47^, ^48^, ^49^ ^]^ These findings provide atomic‐level evidence for the formation of well‐defined Zn‐N_3_O moieties within the S‐CNS and S‐CNT frameworks, confirming the successful construction of single‐atom catalytic sites with customized electronic structures on 1D and 2D C_3_N_4_ structures via a structurally adaptive synthetic approach. The identified coordination geometry, combining S─C, Zn─N, and Zn─O bonds, is expected to create an asymmetric electronic environment.

### 2.2 Activity of S‐CNS‐Zn and S‐CNT‐Zn for H_2_O_2_ Evolution

**Figure 3:**
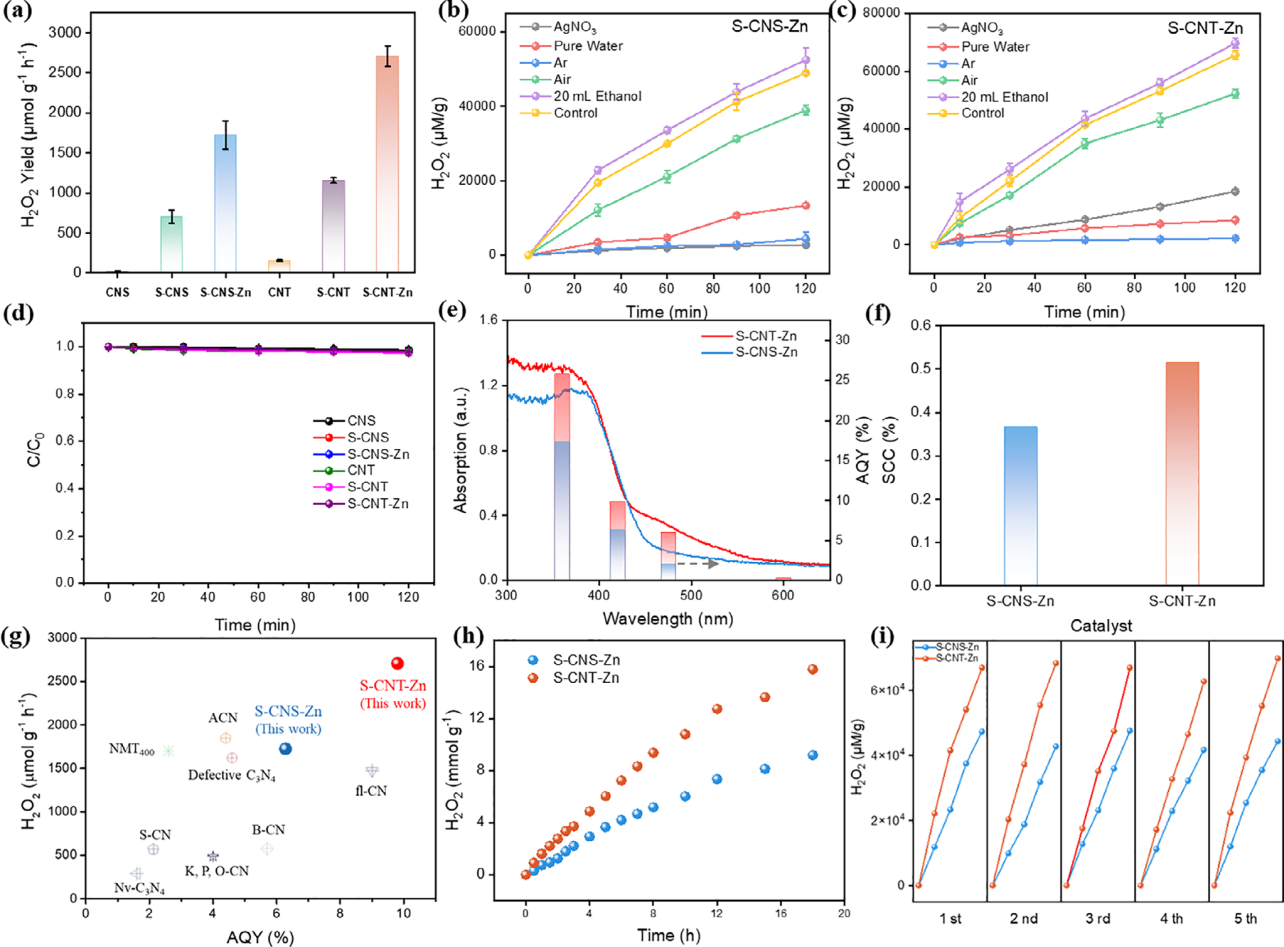
a) Photocatalytic H_2_O_2_ production activity of CNS, CNT, S‐CNS, S‐CNT, S‐CNS‐Zn, and S‐CNT‐Zn. (Reaction condition: 2.5 mg catalyst in 50.0 mL 20 vol% EtOH, λ >420 nm). Activity comparison of b) S‐CNS‐Zn and c) S‐CNT‐Zn in different conditions. d) H_2_O_2_ decomposition behaviors in the presence of six various catalysts under dark conditions. e) AQY (%) and f) SCC (%) of S‐CNS‐Zn and S‐CNT‐Zn. g) Activity comparison of S‐CNS‐Zn and S‐CNT‐Zn with reference samples. h) Time‐dependent H_2_O_2_ evolution of S‐CNS‐Zn and S‐CNT‐Zn. i) Cycling tests of S‐CNS‐Zn and S‐CNT‐Zn for photocatalytic H_2_O_2_ production.

Under visible light irradiation, we evaluated the photocatalytic H_2_O_2_ production performance in an oxygenated ethanol–water system (**Figure** **3**a), where ethanol served as the electron donor and water was the proton source,^[^ ^50^, ^51^, ^52^ ^]^ with H_2_O_2_ quantified by iodometry.^[^ ^53^, ^54^ ^]^ Figure 3a shows that the original CNS had a lower photocatalytic H_2_O_2_ production rate of ≈23.95 µmol g^−1^ h^−1^ than CNT ≈154.68 µmol g^−1^ h^−1^, although CNS has an observed higher crystallinity (Figure S9, Supporting Information). This fact was due to the superior 1D morphology of CNT, which may facilitate the fast and long‐distance transport of photogenerated carriers.^[^ ^7^ ^]^ Moreover, comparative analysis revealed that sulfur modification and zinc single‐atom loading significantly enhanced the photocatalytic H_2_O_2_ production activity compared with pristine C_3_N_4_, demonstrating the crucial role of asymmetric active sites in accelerating H_2_O_2_ generation. Notably, S‐CNT‐Zn exhibited the highest activity at 2708 µmol g^−1^ h^−1^, which was 17.5 times that of CNT and 2.34 times that of S‐CNT, whereas the activity of S‐CNS‐Zn was 71.8 times that of CNS and 2.44 times that of S‐CNS (Figure 3a). In contrast to the modest 6.20 fold enhancement in the H_2_O_2_ production rate achieved by a Zn single‐atom catalyst with a Zn‐N_3_O configuration,^[^ ^25^ ^]^ the highly asymmetric S sites and Zn‐N_3_O configurations in S‐CNS‐Zn and S‐CNT‐Zn exhibit an efficiently synergistic catalytic effect. After S doping, we observed significant differences in the performance improvement of CNS and CNT. The diminished performance enhancement of S‐CNT relative to S‐CNS after S doping may be attributed to the locally symmetric charge distribution caused by S substituting N_3C_ in CNT, which led to the partial coupling of charge carriers.^[^ ^24^ ^]^

To systematically assess environmental factors affecting photocatalytic H_2_O_2_ production, we examined the impacts of pH adjustment and anion introduction (0.2 mmol L^−1^ NaCl, NaHCO_3_, and Na_2_SO_4_) on catalytic performance. As shown in Figure S17 (Supporting Information), the introduced anions (Cl^−^, HCO_3_^−^, and SO_4_^2−^) exhibited negligible effects on H_2_O_2_ generation activity for S‐CNS‐Zn and S‐CNT‐Zn. Both catalysts maintained strong activity across a wide pH range (3.0–9.0), with observed activity reduction under strongly alkaline conditions (pH >9.0) likely attributed to insufficient proton availability for the ORR.^[^ ^50^ ^]^ These results demonstrated the catalysts’ remarkable environmental adaptability. Moreover, as shown in Figure 3b,c, with the addition of electron sacrificial agent AgNO_3_ and Ar atmosphere, S‐CNS‐Zn and S‐CNT‐Zn hardly produced H_2_O_2_, which indicated that the production of H_2_O_2_ on the catalyst was a photocatalytic O_2_ reduction process driven by visible light.^[^ ^55^ ^]^ Additionally, S doping and Zn loading may alter the zeta potential, affecting the catalyst's activity.^[^ ^50^ ^]^ The surface zeta potentials of S‐CNS, S‐CNS‐Zn, and S‐CNT, S‐CNT‐Zn were significantly lower than those of CNS and CNT (Figure S18, Supporting Information). In general, a lower zeta potential favors the binding of O_2_ and protons, promoting the production of hydrogen peroxide through the ORR.^[^ ^50^, ^55^ ^]^ The S modification sites and Zn single atoms disrupt the symmetry of the original C_3_N_4_, changing the charge distribution and affecting the electronic properties of C_3_N_4_.^[^ ^7^, ^56^ ^]^

The high tolerance of H_2_O_2_ to the photocatalyst is also important to realize the high‐speed production and stable storage of H_2_O_2_. Therefore, we tested the samples for H_2_O_2_ decomposition. It hardly catalyzed the decomposition of H_2_O_2_ under illumination (Figure 3d). Under monochromatic light irradiation, the apparent quantum yield (AQY) for photocatalytic H_2_O_2_ production was further evaluated (Figure 3e). The trend of AQY with wavelength matched well with the light absorption curves of S‐CNS‐Zn and S‐CNT‐Zn. Notably, the AQY of S‐CNT‐Zn reached 9.88% at 420 nm and 6.02% at 475 nm, whereas that of S‐CNS‐Zn reached 6.28% at 420 nm and 2.03% at 475 nm. Correspondingly, the solar‐to‐chemical conversion (SCC) efficiencies of S‐CNS‐Zn and S‐CNT‐Zn were as high as 0.37% and 0.52%, respectively (2.5 mg of catalyst, Figure 3f). These values indicated that the charge redistribution and alteration in the electronic band structure caused by the multiple asymmetric catalytic sites in S‐CNS‐Zn and S‐CNT‐Zn resulted in their overall superior performance compared with previously reported g‐C_3_N_4_‐based photocatalytic systems (Figure 3g; Table S4, Supporting Information). For S‐CNS‐Zn and S‐CNT‐Zn, after continuous reaction under visible light irradiation for 18 h, the accumulated H_2_O_2_ concentration reached 9.19 and 15.80 mmol g^−1^ (Figure 3h). These findings demonstrated their good stability, which was further confirmed by the stable production of H_2_O_2_ in the cycling tests (Figure 3i). After the five cycling tests, the crystalline structures of S‐CNS‐Zn and S‐CNT‐Zn remained almost unchanged (Figure S19, Supporting Information). Therefore, S‐CNS‐Zn and S‐CNT‐Zn exhibited good activity and structural stability in photocatalytic H_2_O_2_ production via O_2_ reduction.

### 2.3 Spectra‐Based Mechanism of S‐CNS‐Zn and S‐CNT‐Zn

**Figure 4:**
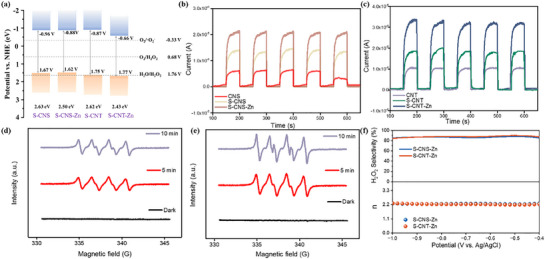
a) Illustration of the band structures of S‐CNS, S‐CNS‐Zn, S‐CNT, and S‐CNT‐Zn. Transient photocurrent response curves of b) CNS, S‐CNS and S‐CNS‐Zn, and c) CNT, S‐CNT, and S‐CNT‐Zn, respectively. EPR test of d) S‐CNS‐Zn and e) S‐CNT‐Zn under dark and light conditions. f) The H_2_O_2_ selectivity and the electron transfer numbers (n) for S‐CNS‐Zn and S‐CNT‐Zn by RRDE (KOH, 0.1 m).

Spectroscopic investigations were conducted to elucidate the charge separation and transfer dynamics during photocatalytic O_2_‐to‐H_2_O_2_ conversion. UV–vis diffuse reflectance spectroscopy (Figures S20 and S21, Supporting Information) revealed a distinct redshift in the absorption edge of S‐CNS‐Zn and S‐CNT‐Zn compared with S‐CNS and S‐CNT, respectively. Bandgap calculations based on the Kubelka–Munk transformation, derived from (αhν)^1/2^ versus hν plots, yielded values of 2.62 (S‐CNT), 2.43 (S‐CNT‐Zn), 2.63 (S‐CNS), and 2.50 eV (S‐CNS‐Zn). Mott‐Schottky plots (Figure S22, Supporting Information) and UV photoelectron spectroscopy (UPS, Figure S23, Supporting Information) were measured to determine the electronic band structure (**Figure** **4**a), revealing that the reduction potentials of O_2_/•O_2_^•^ and O_2_/H_2_O_2_ reside within the mid‐gap states, thermodynamically validating the feasibility of the ORR.^[^ ^57^ ^]^ Furthermore, the introduction of multiple asymmetric sites (N, O, S, and Zn) effectively reduced the bandgap of C_3_N_4_, significantly enhancing the light‐harvesting capabilities of S‐CNT‐Zn and S‐CNS‐Zn (Figure 4a). This conclusion was further supported by photoluminescence (PL) spectroscopy (Figure S24, Supporting Information), which showed a gradual attenuation of PL signal intensity from CNS and CNT to S‐CNS and S‐CNT, and finally to S‐CNS‐Zn and S‐CNT‐Zn, respectively. The systematic PL quenching demonstrated that the multiple asymmetric active sites effectively suppressed radiative recombination and achieved efficient charge separation. This phenomenon may be attributed to the multiple asymmetric active sites that promote charge redistribution and enhance the spatial separation of electrons and hole trapping centers, thereby promoting the separation of photogenerated carriers. Notably, transient photocurrent measurements (Figure 4b,c) showed that S‐CNS‐Zn and S‐CNT‐Zn with multiple asymmetric active sites exhibited the highest photocurrent density in each series of C_3_N_4_‐based catalysts, surpassing their counterparts and providing direct evidence for improved charge carrier separation and transfer.^[^ ^58^ ^]^

To elucidate the critical oxygen intermediates in the O_2_ reduction pathway, we performed electron spin resonance (ESR) spectroscopy using 5,5‐dimethyl‐1‐pyrroline N‐oxide (DMPO) as a spin‐trapping agent for superoxide radicals (•O_2_^−^).^[^ ^59^ ^]^ Under visible light irradiation, S‐CNS‐Zn and S‐CNT‐Zn exhibited characteristic ESR signals corresponding to DMPO‐•O_2_^−^ adducts, whose intensity increased with prolonged illumination (Figure 4d,e).^[^ ^60^ ^]^ This time‐dependent spectral evolution provides direct evidence that i) photogenerated electrons efficiently reduced adsorbed O_2_ to form •O_2_^−^ intermediates, and ii) H_2_O_2_ production occurred via a sequential two‐electron (2e−) reduction mechanism, with •O_2_^−^ acting as the key intermediate. This finding was consistent with the results of the quenching experiments. To further demonstrate the significance of •O_2_^−^ in the formation of H_2_O_2_, nitroblue tetrazolium (NBT), a scavenger of •O_2_^−^, was introduced into the reaction solution.^[^ ^61^ ^]^ Upon the addition of NBT, the activity of H_2_O_2_ production by S‐CNS‐Zn and S‐CNT‐Zn was markedly diminished (Figure S25, Supporting Information), and the reaction solution turned deep purple due to the reduction product of •O_2_^−^ reacting with NBT.^[^ ^62^ ^]^ This outcome further emphasized that H_2_O_2_ production proceeded via a sequential 2e^−^ reduction mechanism. The H_2_O_2_ selectivity and the electron transfer number during the oxygen reduction reaction (ORR) for S‐CNS‐Zn and S‐CNT‐Zn, respectively, were quantitatively evaluated using rotating ring‐disk electrode (RRDE) measurement (Figure 4f). Within the potential window of −1.0 to −0.4 V (vs Ag/AgCl), S‐CNS‐Zn and S‐CNT‐Zn exhibited exceptional H_2_O_2_ selectivity exceeding 86% and 88%, with the electron transfer numbers approaching 2.3 and 2.2, respectively. These electrochemical characterizations provide compelling evidence that both catalysts primarily proceed via the 2 e^−^ ORR pathway conducive to H_2_O_2_ formation. The above results indicated that the significant enhancement in the photocatalytic activity of S‐CNT‐Zn and S‐CNS‐Zn was attributed to the precisely designed asymmetric active sites composed of sulfur dopants and Zn─N_3_O coordination centers. This unique atomic configuration alters their electronic band structure, narrows the bandgap, enhances light absorption capabilities, optimizes the charge distribution, and promotes the separation of photogenerated carriers, thereby enhancing the efficiency of photocatalytic H_2_O_2_ synthesis via the 2e^−^ oxygen reduction pathway (2e^−^‐ORR).

### 2.4 Theoretical Calculations

Density functional theory (DFT) calculations were also performed to reveal the internal mechanism of superior catalytic performance in S‐CNS‐Zn and S‐CNT‐Zn. To elucidate the optimal spatial configuration between the S atom and Zn─N_3_O center, we systematically investigated three distinct models with varying relative distances between S and Zn (Figure S26, Supporting Information).^[^ ^25^ ^]^ Key computational findings demonstrated that the system's total energy increased monotonically with growing S─Zn separation distance (Figure S27, Supporting Information). This result suggested that the Zn single atom exhibited a strong thermodynamic preference for coordinating with the N site closest to the S center. The likely reason for this preference is that the pronounced electron‐rich state on the N atoms near the S dopants in S‐CNS and S‐CNT (Figure S28, Supporting Information) created a favorable electronic environment for Zn binding.

These results provide theoretical evidence that sulfur incorporation preferentially occurred at N_2C_ or N_3C_ positions in immediate proximity to Zn─N_3_O centers. Subsequently, to further determine the optimal oxygen coordination geometry in the Zn─N_3_O structure relative to the predetermined sulfur sites, we conducted a systematic computational investigation by evaluating four distinct oxygen‐doped Zn─N_3_O configurations (Figure S29, Supporting Information). The first‐principle in calculations revealed that configuration III (Figure S29a,b, Supporting Information) exhibited the most thermodynamically favorable formation energy (E_f_ = −3.40 and 0.75 eV) compared with the other configurations, with key structural advantages.^[^ ^35^ ^]^

**Figure 5:**
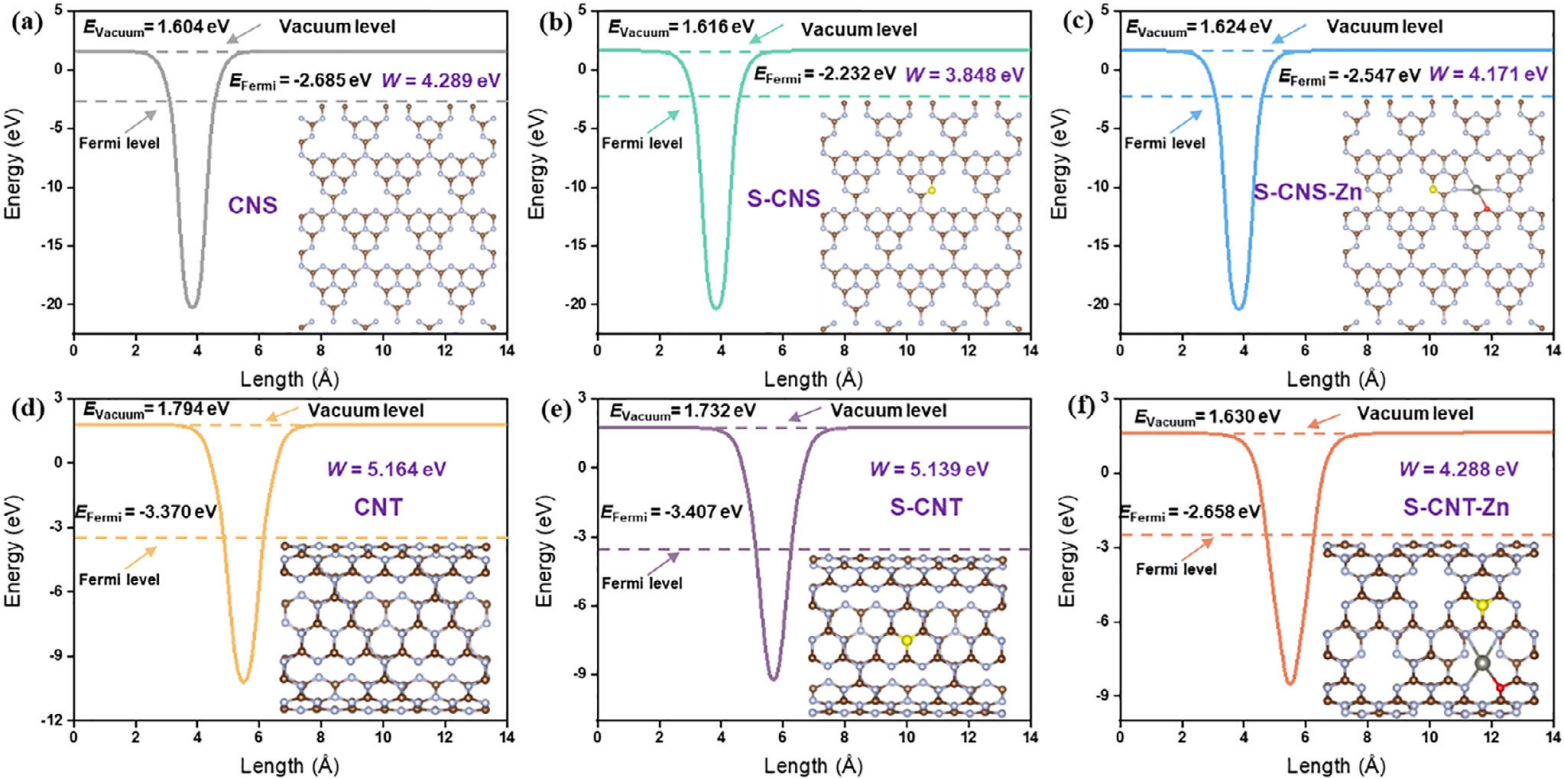
Work functions of a) CNS, b) S‐CNS, c) S‐CNS‐Zn, d) CNT, e) S‐CNT, and f) S‐CNT‐Zn, respectively.

Thus, configuration III was selected as the optimized model for simulating S‐CNS‐Zn and S‐CNT‐Zn. The photogenerated charge separation and migration efficiency within the conjugated planes of 1D and 2D structures play a pivotal role in determining photocatalytic performance. Our systematic work function calculations (Φ) revealed a remarkable electronic structure modulation. Φ is defined as the minimum energy required for electrons to escape from the material surface to the vacuum, directly reflecting the material's ability to donate electrons to adsorbed species (such as O_2_ in this study).^[^ ^63^ ^]^ In the series of 1D C_3_N_4_‐based materials, pristine CNT exhibited Φ = 5.164 eV (**Figure** **5**d), whereas sulfur doping induced a slight reduction to 5.139 eV (Figure 5e). The synergistic incorporation of the S site and Zn‐N_3_O center led to a dramatic decrease to 4.288 eV (Figure 5f). The series of 2D C_3_N_4_‐based materials indicated that the presence of S with Zn─N_3_O in S‐CNS‐Zn required slightly more energy than S‐CNS alone (Figure 5a–c), which may stem from electron attraction by oxygen sites within the 2D conjugated plane.^[^ ^35^ ^]^ This is corroborated by the charge‐enriched state of oxygen in the ground‐state S‐CNS‐Zn (Figure S30, Supporting Information). The charge transfer between the adsorbed intermediates and the material is closely related to the Φ. On one hand, the unique coordination environment—a combination of multiple S, Zn, N, and O sites—reduces the surface Φ of the material. A smaller Φ lowers the energy barrier for electrons to escape into the vacuum, facilitating the transfer of electrons from the catalyst surface to the adsorbed oxygen molecules.^[^ ^64^ ^]^ On the other hand, the number of transferred electrons is significantly correlated with the work function difference (ΔW) between the catalyst and the adsorbate. Figure S31 (Supporting Information) shows that the lower the ΔW value is, the more electrons are transferred from the catalyst surface to ^*^OO. A lower ΔW value also indicates a lower Φ of the catalyst before adsorption, which confirms that electrons in S‐CNS‐Zn/S‐CNT‐Zn are more likely to spill over from the catalyst surface and transfer to the adsorbed ^*^OO intermediates, ultimately enhancing the 2e^−^‐ORR activity.^[^ ^65^ ^]^

**Figure 6:**
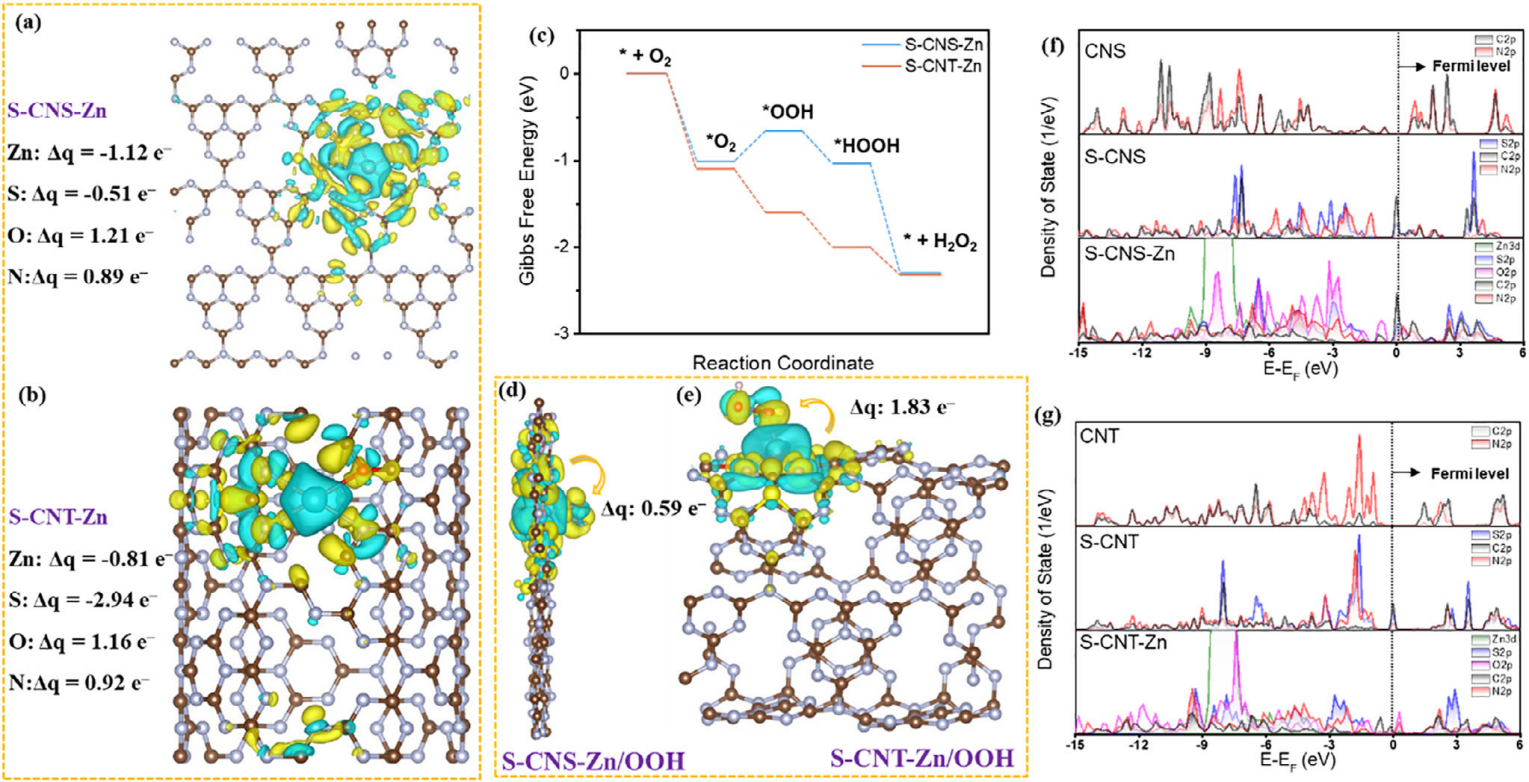
Differential charge density of a) S‐CNS‐Zn and b) S‐CNT‐Zn models and corresponding charge change of Zn centers. c) Free energy diagram of 2e^−^ O_2_ reduction on Zn sites within different models. Differential charge density of d) S‐CNS‐Zn/OOH and e) S‐CNT‐Zn/OOH models and the corresponding charge accumulated in ^*^OOH intermediate. Calculated partial density of states (PDOS) for f) S‐CNS‐Zn and g) S‐CNT‐Zn models. In Figure 6a,b,d,e, yellow and blue areas represent electron accumulation and electron depletion, respectively.

**Figure** **6**a,b displays the charge density difference plots generated, with yellow regions indicating electron accumulation and blue regions representing electron depletion. In S‐CNS‐Zn (*Δq* = −0.51 e^−^) and S‐CNT‐Zn (*Δq* = −2.94 e^−^), sulfur atoms exhibited significant electron loss, which was much higher than that in the standalone sulfur‐doped systems, as shown in Figure S28 (Supporting Information) (S‐CNS (*Δq* = −0.44 e^−^) and S‐CNT (*Δq* = −0.07 e^−^)). Thus, the incorporation of Zn–N_3_O further disrupted the symmetry of the triazine repeat unit and increased the charge transfer of sulfur. The distinct charge density differences between S/Zn (electron‐depleted regions) and the neighboring N/O (electron‐enriched regions) created a gradient distribution that disrupted the homogeneity of the electron cloud in the symmetric catalytic centers of C_3_N_4_, which helped improve the delocalization of π‐electrons and promote the spatial separation of charge carriers in C_3_N_4_.^[^ ^7^ ^]^ The reduced electron transfer of sulfur in S‐CNT compared with S‐CNS (Figure S28, Supporting Information) corresponded with the significantly weaker performance enhancement of S‐CNT compared with S‐CNS observed in catalytic experiments (Figure 3a). The localized symmetric charge distribution in S‐CNT, induced by sulfur substitution at N_3C_ sites, led to partial coupling of charge carriers. This electronic configuration explained why S‐CNT demonstrated lower sulfur electron transfer and less pronounced performance enhancement compared with S‐CNS (Figure 3a; Figure S28, Supporting Information).

Additionally, projected density of states (PDOS) analysis was performed for different samples to investigate the influence of the introduction of multiple asymmetric sites on the electronic structure of C_3_N_4_‐based materials. The PDOS analysis of S‐CNS and S‐CNT revealed that the introduction of S sites led to a redistribution of the electronic structure, which caused the emergence of a new intermediate state gap near the bottom of the conduction band (CB), corresponding to the peak appearing around the Fermi level (Figure 6f,g). This change altered the band structures of CNS and CNT, which not only narrowed their bandgaps but also enhanced their light absorption capacity.^[^ ^35^ ^]^ The limited overlapping electronic states between sulfur (blue) and carbon (gray) near the Fermi level (E‐E_f_ = 0) reflected restricted charge delocalization characteristics (Figure 6f,g). By contrast, the PDOS of S‐CNS‐Zn and S‐CNT‐Zn near the Fermi level revealed extended orbital overlap regions between S and C/N (Figure 6f,g). The PDOS of Zn showed substantial overlap with nitrogen (red) and oxygen (pink) states in the E‐E_f_<0 region, demonstrating strong interactions (e.g., chemical bonding) through orbital hybridization (Figure 6f,g). The broadened spectrum of orbital hybridization and overlapping peak regions confirmed the redistributed system charge and enhanced electronic delocalization.^[^ ^23^, ^66^, ^67^ ^]^ Notably, in S‐CNS‐Zn and S‐CNT‐Zn, the unfilled orbitals of Zn made a significant contribution to the CB (Figure S32a, Supporting Information). This not only drove the band offset but also facilitated the migration of electrons from the valence band (VB) to the CB. In contrast, N and O elements exerted a more prominent influence on the VB (Figure S32c,d, Supporting Information), enabling N and O atoms to act as effective electron donors.^[^ ^35^ ^]^ Specifically, the unfilled orbitals of Zn and the partial electron contributions from N and O elements remained the primary contributors to the CB and VB, respectively.^[^ ^68^ ^]^ In addition, PDOS results indicated that S atoms made important contributions to both the composition of the VB and CB (Figure S32b, Supporting Information), allowing S atoms to function as effective electron donors and acceptors.^[^ ^68^ ^]^ Therefore, under external energy excitation, electrons at N and O sites were preferentially transferred to the unfilled orbitals of Zn sites.^[^ ^35^, ^68^ ^]^ A portion of the valence electrons transferred to S can further migrate to single Zn atoms through the conjugated framework under the influence of the internal potential field.^[^ ^69^ ^]^ These multiple carrier transfer pathways in parallel reduced the electron transport demand on a single channel, thereby significantly decreasing recombination caused by local charge accumulation.^[^ ^57^, ^68^ ^]^ Moreover, the direction of electron transfer is further confirmed by investigation of the changes in the electronic structure of each active center before and after ^*^OOH adsorption. Bader charge calculations exhibited in Figure S30 (Supporting Information) indicate that the presence of multiple asymmetric sites causes N and O sites to lose more electrons during the catalytic reaction, while Zn sites, after receiving the transferred electrons, further transfer them to the adsorbed intermediates, exhibiting a more electron‐deficient state.^[^ ^70^ ^]^

To elucidate the thermodynamic distinctions in reaction pathways, we systematically investigated the key intermediates involved in 2e^−^ oxygen reduction through free energy (*ΔG*) calculations (Figure 6c). The reaction proceeded via sequential steps: O_2_ adsorption → ^*^OO → ^*^OOH → ^*^HOOH → H_2_O_2_ desorption. *ΔG* of O_2_ adsorption on S‐CNT‐Zn was lower than that on S‐CNS‐Zn, indicating that O_2_ adsorption was more favorable in the former case than in the latter. Simultaneously, DFT calculation revealed that S‐CNT‐Zn possessed a greater ability to facilitate the deprotonation of H_2_O, yielding a higher number of proton sources compared with S‐CNS‐Zn (Figure S33, Supporting Information).^[^ ^71^ ^]^ The above results were aligned with the prior results derived from zeta potential tests (Figure S18, Supporting Information), demonstrating that a low surface potential facilitated the binding of O_2_ and protons. Furthermore, as illustrated in Figure S34 (Supporting Information), calculations on the key intermediates in the competitive 4e^−^ ORR pathway revealed that the free energy difference for ^*^OOH conversion to H_2_O_2_ (*ΔG*_S1_ = −1.64 eV; *ΔG*_T1_ = −0.72 eV) is lower than those for ^*^OOH dissociation to ^*^O (*ΔG*_S2_ = −1.42 eV; *ΔG*_T2_ = −0.61 eV). The selectivity ratios (*ΔG*_S1_/*ΔG*_S2_ = 1.15 and *ΔG*_T1_/*ΔG*_T2_ = 1.18) are greater than unity, confirming that ^*^OOH adsorbed on S‐CNS‐Zn and S‐CNT‐Zn preferentially undergoes hydrogenation to form H_2_O_2_ rather than cleaving to ^*^O.^[^ ^72^ ^]^ This aligns with the high 2e^−^ ORR selectivity observed in RRDE experiments (Figure 4f).

For S‐CNS and S‐CNT, the formation of ∗OO was the rate‐determining step. However, upon further incorporation of Zn‐N_3_O, S‐CNS‐Zn and S‐CNT‐Zn exhibited significantly enhanced affinity for O_2_ adsorption (Figures S35 and S36, Supporting Information), indicating that O_2_ was readily bound at the Zn active sites. This strong adsorption ability could be attributed to the enhanced π‐electron delocalization resulting from the incorporation of additional Zn, O, and N active sites. Bader charge analysis shows that the ^*^OO intermediate in S‐CNS‐Zn and S‐CNT‐Zn accepts 0.57 and 0.58 e^−^ from the catalyst, respectively (in contrast, the values are 0.36 and 0.15 e^−^ in S‐CNS and S‐CNT, as shown in Table S3, Supporting Information). This is a direct result of enhanced electron delocalization, enabling more efficient charge transfer to the adsorbed O_2_.^[^ ^73^ ^]^ Additionally, simulations of Gibbs free energy changes during the process revealed that the energy barrier for the conversion from ^*^OO to ^*^OOH by S‐CNS‐Zn and S‐CNT‐Zn was significantly lower than that by S‐CNS and S‐CNT (Figure S35, Supporting Information), suggesting that O_2_ adsorbed on the catalysts with multiple asymmetric sites was easily activated into ^*^OOH. The reason for the reduced energy barrier for ^*^OOH intermediate formation can be attributed to the highly asymmetric structure (Zn‐N_3_O coordination combined with S doping), which induces extensive electron delocalization. Enhanced electron delocalization promotes electron transfer to ^*^O_2_, weakens the O═O bond, and thereby lowers the energy barrier for ^*^OOH formation. DFT calculations reveal that the O═O bond lengths in ^*^OO adsorbed on S‐CNS‐Zn and S‐CNT‐Zn are elongated to 1.47 and 1.33 Å, respectively (in contrast, the bond length is 1.21 Å in free O_2_, 1.28 Å in S‐CNS, and 1.25 Å in S‐CNT, Table S3, Supporting Information). Moreover, Bader charge analysis and differential charge density analysis revealed that, compared with S‐CNS (0.49 e^−^) and S‐CNT (0.183 e^−^) (Figure S37, Supporting Information), there was a more pronounced electron transfer from S‐CNS‐Zn (0.59 e^−^) and S‐CNT‐Zn (1.83 e^−^) to the intermediate ^*^OOH (Figure 6d,e; Figure S38, Supporting Information). Combined with PDOS analysis, the numerous carrier transfer paths generated by the multiple asymmetric sites of S/Zn and N/O promoted electron accumulation on ^*^OOH, which facilitated the reduction of O_2_ to H_2_O_2_.

## 3 Conclusion

In summary, we developed a structurally adaptive strategy to construct multiple asymmetric catalytic sites by integrating Zn–N_3_O centers into sulfur‐doped C_3_N_4_. The optimized S‐CNS‐Zn and S‐CNT‐Zn catalysts exhibited exceptional H_2_O_2_ generation rates of 1724 and 2708 µmol g^−1^ h^−1^, respectively, under visible light in a sacrificial system. Our findings revealed that the superior activity of the catalyst may originate from its highly asymmetric structure, which possessed a broadened visible‐light spectrum. The asymmetric distribution of N, S, O, and Zn elements within the triazine repeating units disrupted the uniformity of the electron cloud in the C_3_N_4_ catalytic centers, enhancing electron delocalization and creating multiple parallel atomic‐scale charge transfer channels. These features collectively enabled the effective separation and transfer of electron–hole pairs, enhanced O_2_ adsorption, and the indirect two‐step 2e^−^‐ORR. Notably, this approach demonstrated consistent site distributions throughout 1D and 2D C_3_N_4_ frameworks, highlighting its potential for the design and fabrication of high‐performance photocatalysts beyond H_2_O_2_ synthesis. This work presents novel insights into symmetry‐breaking strategies for energy conversion technologies and offers ideas for tailored catalytic systems in sustainable chemistry.

## Conflict of Interest

The authors declare no conflict of interest.

## Supporting information



Supporting Information

## Data Availability

The data that support the findings of this study are available from the corresponding author upon reasonable request.
