# Supplementary material for: Construction of Multiple Asymmetric Catalytic Sites on Carbon Nitrides Toward Efficient Solar Hydrogen Peroxide Production
Source: Adv Sci (Weinh). 2025 Sep 16;12(45):e13453. doi: 10.1002/advs.202513453 (PMC12677617; doi:10.1002/advs.202513453)
Supplement: Supplementary file 1 — Supporting Information [file ADVS-12-e13453-s001.docx]

**Supporting Information**

**Construction of Multiple Asymmetric Catalytic Sites on Carbon Nitrides toward Efficient Solar Hydrogen Peroxide Production**

Siyu Sun^1^, Feng Gao^2^, Hu Yang^1^*

^1^ State Key Laboratory of Water Pollution Control and Green Resource Recycling, School of the Environment, Nanjing University, Nanjing 210023, P. R. China

^2^ Department of Materials Science and Engineering, Jiangsu Key Laboratory of Artificial Functional Materials, Collaborative Innovation Center of Advanced Microstructures, College of Engineering and Applied Sciences, Nanjing University, Nanjing 210023, P. R. China

——————————————————

* Corresponding author. E-mail: yanghu@nju.edu.cn

**Part 1 Experimental Section**

**Materials and chemicals**

All chemicals used in this work are analytical grade and utilized without further purification. Urea (CO(NH_2_)_2_, AR), melamine (C_3_H_6_N_6_, AR), sodium hydrogen sulfite (NaHSO_3_, AR), zinc acetate dihydrate (Zn(OAc)_2_•2H_2_O, 99.9% metals basis), N,N-dimethylformamide (DMF, AR), ethanol (EtOH, AR), sodium sulfate (Na_2_SO_4_, AR), potassium iodide (KI, AR), potassium hydrogen phthalate (KC_8_H_5_O_4_, AR), silver nitrate (AgNO_3_, AR) and sodium sulfate (Na_2_SO_4_,AR) nitrotetrazolium blue chloride (NBT, 98%) were purchased from Aladdin Industrial Co., Ltd.

**Synthesis of CNS**

10.0 g urea and 1.0 g melamine were evenly ground in an agate mortar, and then placed in a muffle furnace. The temperature was raised to 550℃ at a rate of 2℃/min for 4 h.

**Synthesis of CNT**

5.0 g of urea and 0.5 g of melamine were evenly ground in an agate mortar, and then placed in a tube furnace. In N_2_ atmosphere, the temperature was raised to 570℃ at a rate of 3℃/min and the temperature was maintained for 1.5 h.

**Synthesis of S-CNS**

10.0 g urea, 1.0 g melamine and a desired amount of NaHSO_3_ were evenly ground in an agate mortar, and then placed in a muffle furnace. The temperature was raised to 550℃ at the rate of 2℃/min, and the temperature was kept for 4 h. When the specific amount of NaHSO_3_ was 20.0 mg, 40.0 mg, 80.0 mg and 160.0 mg, the obtained was S-CNS-1, S-CNS-2 S-CNS-3 and S-CNS-4, respectively. S-CNS-2 was the optimal one with the highest H_2_O_2_ generation activity (SI Figs. S1a and 1b) and was hereinafter referred to as S-CNS for short. The mass of the S-CNS-2 obtained is 3.64 g.

**Synthesis of S-CNT**

5.0 g urea, 0.5 g melamine and a desired amount of NaHSO_3_ were evenly ground in an agate mortar, and then placed in a tube furnace. In N_2_ atmosphere, the temperature was raised to 570℃ at the rate of 3℃/min, and the temperature was kept for 1.5 h. When the specific amount of NaHSO_3_ was 10.0 mg, 20.0 mg, 40.0 mg, 80.0 mg, the obtained was S-CNT-1, S-CNT-2, S-CNT-3 and S-CNT-4, respectively. S-CNT-2 was the optimal one with the highest H_2_O_2_ generation activity (SI Figs. S1a and 1b) and was hereinafter referred to as S-CNT for short. The mass of the S-CNT-2 obtained is 0.789 g.

**Synthesis of S-CNS-Zn**

A desired amount of Zn(OAc)_2_ was dissolved in 50.0 mL DMF solution. 40.0 mg of S-CNS was mixed with 10.0 mL of ethanol and added to DMF solution, and stirred at 70℃ for 2 h. After filtering and then drying at 50℃ overnight, the white powder was put into a tube furnace and heated to 400℃ at a rate of 5℃/min in the Ar atmosphere for 60 min. When the specific amount of Zn(OAc)_2_ was 0.5 mg, 1.1 mg, 2.1 mg, 5.4 mg, 8.6 mg and 10.7 mg, the obtained was S-CNS-Zn-1, S-CNS-Zn-2, S-CNS-Zn-3, S-CNS-Zn-4, S-CNS-Zn-5 and S-CNS-Zn-6, respectively. S-CNS-Zn-2 was the optimal one with the highest H_2_O_2_ generation activity (SI Figs. S1c and 1d) and was hereinafter referred to as S-CNS-Zn for short. The mass of the S-CNS-Zn-2 obtained is 37.2 mg.

**Synthesis of S-CNT-Zn**

A desired amount of Zn(OAc)_2_ were dissolved in 50.0 mL DMF solution. 40.0 mg of S-CNT was mixed with 10.0 mL of ethanol and added to DMF solution, and stirred at 70℃ for 2 h. After filtering and then drying at 50℃ overnight, the white powder was put into a tube furnace and heated to 400℃ at a rate of 5℃/min in the Ar atmosphere for 60 min. When the specific amount of Zn(OAc)_2_ was 0.5 mg, 1.1 mg, 2.1 mg, 5.4 mg, 8.6 mg and 10.7 mg, the obtained was S-CNT-Zn-1, S-CNT-Zn-2, S-CNT-Zn-3, S-CNT-Zn-4, S-CNT-Zn-5 and S-CNT-Zn-6, respectively. S-CNT-Zn-2 was the optimal one with the highest H_2_O_2_ generation activity (SI Figs. S1c and 1d) and was hereinafter referred to as S-CNT-Zn for short. The mass of the S-CNT-Zn-2 obtained is 34.6 g.

**Yield Calculation**

The yield formula of tubular and flake C_3_N_4_ is shown as follows:

$\text{Yield}\left( \text{\%} \right)\text{=}\left( \frac{\text{Actual mass of }\text{C}_{\text{3}}\text{N}_{\text{4}}}{\text{Theoretical mass of }\text{C}_{\text{3}}\text{N}_{\text{4}}} \right)\text{×100\%}$ (E1)

The ideal polycondensation reaction (simplified model) of urea in an air or N_2_ atmosphere is shown as follows:

${\text{6CO(}\text{NH}_{\text{2}}\text{)}}_{\text{2}}\text{→2}\text{C}_{\text{3}}\text{N}_{\text{4}}\text{+}\text{x}\text{NH}_{\text{3}}\text{+yH}_{\text{2}}\text{O}$ (E2)

Since it involves a complex condensation process for polymer generation, the by-products (NH_3_ and H_2_O) are formed in variable proportions and are therefore represented by *x* and *y*. According to the yield formula, the theoretical yield of C_3_N_4_ generated from 10.0 g of urea is 5.11 g.

The ideal reaction equations for the formation of C_3_N_4_ from melamine in an air and N_2_ atmosphere, respectively, are shown as follows:

$\text{2}\text{C}_{\text{3}}\text{H}_{\text{6}}\text{N}_{\text{6}}\text{+3}\text{O}_{\text{2}}\text{→2}\text{C}_{\text{3}}\text{N}_{\text{4}}\text{+x}\text{NH}_{\text{3}}\text{+}\text{yH}_{\text{2}}\text{O}$ (E3)

$\text{2}\text{C}_{\text{3}}\text{H}_{\text{6}}\text{N}_{\text{6}}\text{→2}\text{C}_{\text{3}}\text{N}_{\text{4}}\text{+n}\text{NH}_{\text{3}}\text{+}\text{mH}_{\text{2}}$ (E4)

Since it involves a complex condensation process for polymer generation, the by-products (NH_3_ and H_2_O or NH_3_ and H_2_) are formed in variable proportions and are therefore represented by *x*, *y* and *n*, *m*. According to the yield formula, the theoretical yield of C_3_N_4_ generated from 1.0 g of melamine is 0.730 g.

Since S doping replaces N atoms in C_3_N_4_, the mass increment after S substitution can be calculated. Therefore, the theoretical yield of S-CNS generated from 40 mg of NaHSO_3_, 10 g of urea, and 1.0 g of melamine is 5.85 g. The theoretical yield of S-CNT generated from 20 mg of NaHSO_3_, 5.0 g of urea, and 0.5 g of melamine is 2.920 g.

It was deduced from EXAFS and XPS that the Zn single atoms exist in the form of Zn-N_3_O coordination. Based on this, when 40 mg of S-CNS/S-CNT and 1.1 mg of Zn(OAc)_2_ were used, the theoretical production masses of S-CNS-Zn and S-CNT-Zn were estimated about 40.42 mg.

**Characterizations**

X-ray diffraction (XRD) (X’TRA, Thermo Fisher Scientific, USA) of catalysts were performed in the range of 2θ from 4 to 70° with Cu Kα radiation. The physical morphology and elemental distribution of the as-prepared materials were characterized by scanning electron microscopy (SEM) (Quanta-250 FEG, FEI, USA) and transmission electron microscope (TEM) (JEM-200CX, JEOL, Japan). X-ray photoelectron spectroscopy (XPS) was used to measure the elemental chemical state and valence band energy of materials (PHI-5000 Versa Probe, ULVAC-PHI, Japan). The specified surface areas were obtained using a Brunauer-Emmett-Teller (BET) method (Micromeritics ASAP 2460, USA). The functional groups contained in catalysts were tested by Fourier transform infrared (FTIR) (NEXUS870, NICOLET, USA). UV-vis diffuse reflectance spectra (DRS) (Shimadzu, Kyoto, Japan) tested the light absorption capacity of the sample and was used to calculate the bandgap width. The I-T curve was measured by an electrochemical workstation (CHI-760E, Chen Hua. Shanghai, China). Moreover, the recombination efficiency of photo-generated carriers was tested by Photoluminescence (PL) (Horiba Fluorolog-3-22 type, HORIBA, USA).

**Photochemical measurement**

Transient photocurrent response was conducted on an electrochemical workstation (CHI-760E, Shanghai Chenhua Apparatus Corporation, China) with a standard three-electrode system. Photocatalyst coated on indium tin oxide (ITO) glass was used as working electrode, glass as working electrode, Pt sheet as counter electrode, calomel electrode as reference electrode, and 0.5 mol/L Na_2_SO_4_ solution as electrolyte.

**Evaluation of photocatalytic performances**

Using a photochemical reactor with a 300 W xenon lamp equipped with a 420 nm cut-off filter, the photocatalytic performance of the as-obtained products was evaluated through the generation experiment of H_2_O_2_ under visible light irradiation. 2.5 mg of photocatalyst was mixed in 50.0 mL of solution containing 40.0 mL of deionized water and 10.0 mL of ethanol. Then, O_2_ was bubbled for 30 minutes to reach oxygen saturation. After dark reaction for 30 minutes, the system was exposed to visible light. At a specific time interval, the suspension was collected, filtered by a filter head (0.22 μm, water system), and 1.0 mL of the filtered solution was used to analyze the concentration of H_2_O_2_ (iodometry). Cyclic experiments were conducted to evaluate the stability of the samples.

**AQY measurements**

The apparent quantum yield (AQY) was tested by using a 300-W Xe lamp irradiation equipped with band pass filter of 360 nm, 420 nm and 475 nm. The intensity of the monochromic light was averaged at 5 representative points by using an optical meter (Beijing Perfectlight, model 1918-R). Then, AQY was calculated by the following equation:

$\text{AQY }\left( \text{\%} \right)\text{ = }\frac{\text{(number of }{\text{H}_{\text{2}}\text{O}}_{\text{2}}\text{ production)× 2}}{\text{number of incident photons}}\text{×100}\text{\%}$ (E5)

The number of incident photons is:

$\text{N}_{\text{incident}}\text{=}\frac{\text{Pt}}{\text{hv}}\text{=}\frac{\text{Ptλ}}{\text{hc}}\text{=}\frac{\text{IStλ}}{\text{hc}}$ (E6)

where *I* is the light power intensity (W m^−2^), *S* is the irradiation area (m^2^), *t* is the reaction time (s), *λ* is the wavelength length (m) of the monochromatic light, *h* is Planck’s constant (6.63×10^−34^ m^2^ kg s^−1^), and *c* is the speed of light in free space (3.0 ×10^8^ m s^−1^).

**SCC measurements**

The solar-to-chemical conversion (SCC) efficiency was tested using a solar simulator for irradiation. The SCC efficiency was calculated by the following equation:

$\text{SCC efficiency }\left( \text{\%} \right)\text{=}\frac{\left[ \text{ΔG for }\text{H}_{\text{2}}\text{O}_{\text{2}}\text{ generation }\left( \text{J }\text{mol}^{\text{-1}} \right) \right]\text{×[}\text{H}_{\text{2}}\text{O}_{\text{2}}\text{ formed (mol) }}{\left[ \text{Total input power }\left( \text{W} \right) \right]\text{×[Reaction time(s)]}}\text{×100}$ (E7)

in which, 10.0 mg of catalysts and 40.0 mL of water and 10.0 mL ethanol were put into a top-irradiation reactor attached to a sealed glass system. The free energy for H_2_O_2_ formation was 117 kJ mol^–1^. The overall irradiance of the AM1.5 global spectrum (300−760 nm) was 100 mW cm^−2^, and the irradiated area was 0.273 m^2^. Then, the total input energy was calculated as 2.02 W.

**Ultraviolet photoelectron spectroscopy (UPS) measurements**

The valence band maximum (E_VBM_) of the prepared photocatalyst (*versus* vacuum level) is calculated by the following formula.

$\text{E}_{\text{VBM}}\text{=hv-}\text{E}_{\text{C}}\text{+}\text{E}_{\text{F}}$ (E8)

$\text{E}_{\text{VBM}}\text{(vs. NHE)=}\text{E}_{\text{VBM}}\text{-4.44}$ (E9)

where *hv* is the incident photon energy of 21.22 eV in UPS. Fermi energy level (*E*_F_) and high kinetic energy cutoff value (*E*_C_) are determined by the corresponding tangents in each region.

**RRDE measurements**

The Ag/AgCl (KCl-saturated) electrode was used as the reference electrode, and the graphite rod electrode was used as the counter electrode. The cyclic voltammetry (CV) was performed at the scan rate of 50 mV s^–1^ and the linear scan voltammetry (LSV) was measured at the scan rate of 10 mV s^–1^. KOH (0.1 M) served as the electrolyte. The LSV test was done in N_2_ saturated and O_2_ saturated electrolytes, respectively. For the ORR test, the platinum ring potential of the RRDE was fixed at 0.2 V (vs. Ag/AgCl) and RRDE measurements were performed at a rotation speed of 1600 rpm. The electron transfer number (*n*) and H_2_O_2_ yields were calculated by the following equations.

$\text{H}_{\text{2}}\text{O}_{\text{2}}\text{\%=200×}\frac{\text{I}_{\text{R}}\text{/N}}{\text{I}_{\text{D}}\text{+}\text{I}_{\text{R}}\text{/N}}$*%* (E10)

$\text{n=4×}\frac{\text{I}_{\text{D}}}{\text{I}_{\text{D}}\text{+}\text{I}_{\text{R}}\text{/N}}$ (E11)

Herein, *I*_R_ and *I*_D_ are ring and disk currents, respectively. *N* is the collection efficiency of the ring electrode and is measured to be 0.37.

**X-ray Absorption fine structure spectra (XAFS) measurement**

Zn K-edge analysis was performed with Si (111) crystal monochromators at the BL11B beamlines at the Shanghai Synchrotron Radiation Facility (SSRF) (Shanghai, China). Before the analysis at the beamline, samples were pressed into thin sheets with 1.0 cm in diameter and sealed using Kapton tape film. The XAFS spectra were recorded at room temperature using a 4-channel Silicon Drift Detector (SDD) Bruker 5040. Zn K-edge extended X-ray absorption fine structure (EXAFS) spectra were recorded in transmission mode. Negligible changes in the line-shape and peak position of Zn K-edge XANES spectra were observed between two scans taken for a specific sample. The XAFS spectra of these standard samples (ZnPC, ZnO, and ZnS) were recorded in transmission mode. The spectra were processed and analyzed by the software codes Athena and Artemis.

**Density Functional Theory (DFT) calculations**

The DFT calculations in this work were performed by applying a generalized gradient approximation with the Perdew-Burke-Ernzerhof (PBE) exchange and correlation functional using the “Vienna ab initio simulation package” (VASP 5.4).^[S1, S2]^ We have chosen the projected augmented wave (PAW) potentials to describe the ionic cores and take valence electrons into account using a plane wave basis set with a kinetic energy cutoff of 400 eV.^[S3, S4]^ Partial occupancies of the Kohn−Sham orbitals were allowed using the Gaussian smearing method and a width of 0.1 eV. For the optimization of both geometry and lattice size, the Brillouin zone integration was performed with 1×1×1 *Γ*-centered k-point sampling, and for the density of states calculation, the Brillouin zone integration was performed with 4*2*1 Monkhorst-Pack k-point sampling.^[S5]^ The self-consistent calculations applied a convergence energy threshold of 10^-5^ eV. The equilibrium geometries and lattice constants were optimized with maximum stress on each atom within 0.1 eV Å^-1^. The 15 Å vacuum layer was normally added to the surface to eliminate the artificial interactions between periodic images. The adsorption energy (*E*_ads_) can be defined as follows:

$\text{E}_{\text{ads}}\text{=}\text{E}_{\text{tot}}\text{-(}\text{E}_{\text{s}}\text{+}\text{E}_{\text{c}}\text{)}$ (E12)

where *E*_tot_, *E*_s_, and *E*_c_ depict the total energies of the adsorption complex, the isolated molecule, and the catalyst, respectively.

The Gibbs free energy (*ΔG*) for all PAA activation reactions is defined as follows:

$\text{Δ}\text{G= }\text{Δ}\text{E+ }\text{Δ}\text{ZPE-T}\text{Δ}\text{S}$ (E13)

where *ΔE*, *ΔZPE*, *T*, and *ΔS* are the DFT energy of the adsorption complex, zero-point energy difference, reaction temperature, and entropy difference, respectively.

The formation energies of substitutional doping and interstitial doping can be calculated by the following formulas:

$\text{E}_{\text{f}}\text{=}\text{E}_{\text{a}}\text{-}\text{E}_{\text{b}}\text{-}\text{E}_{\text{i}}$ (E14)

where *E_f_*, *E_a_*, *E_b_* and *E_i_* are doping formation energy, total energy of the material after doping, total energy of the material before doping, energy of the interstitial doped atoms.

Moreover, Bader charge analysis was also applied to this work.^[S6-S9]^ The data of differential charge density was calculated by the Vaspkit.^[S10]^

**Part 2 Supplemental Results**

**
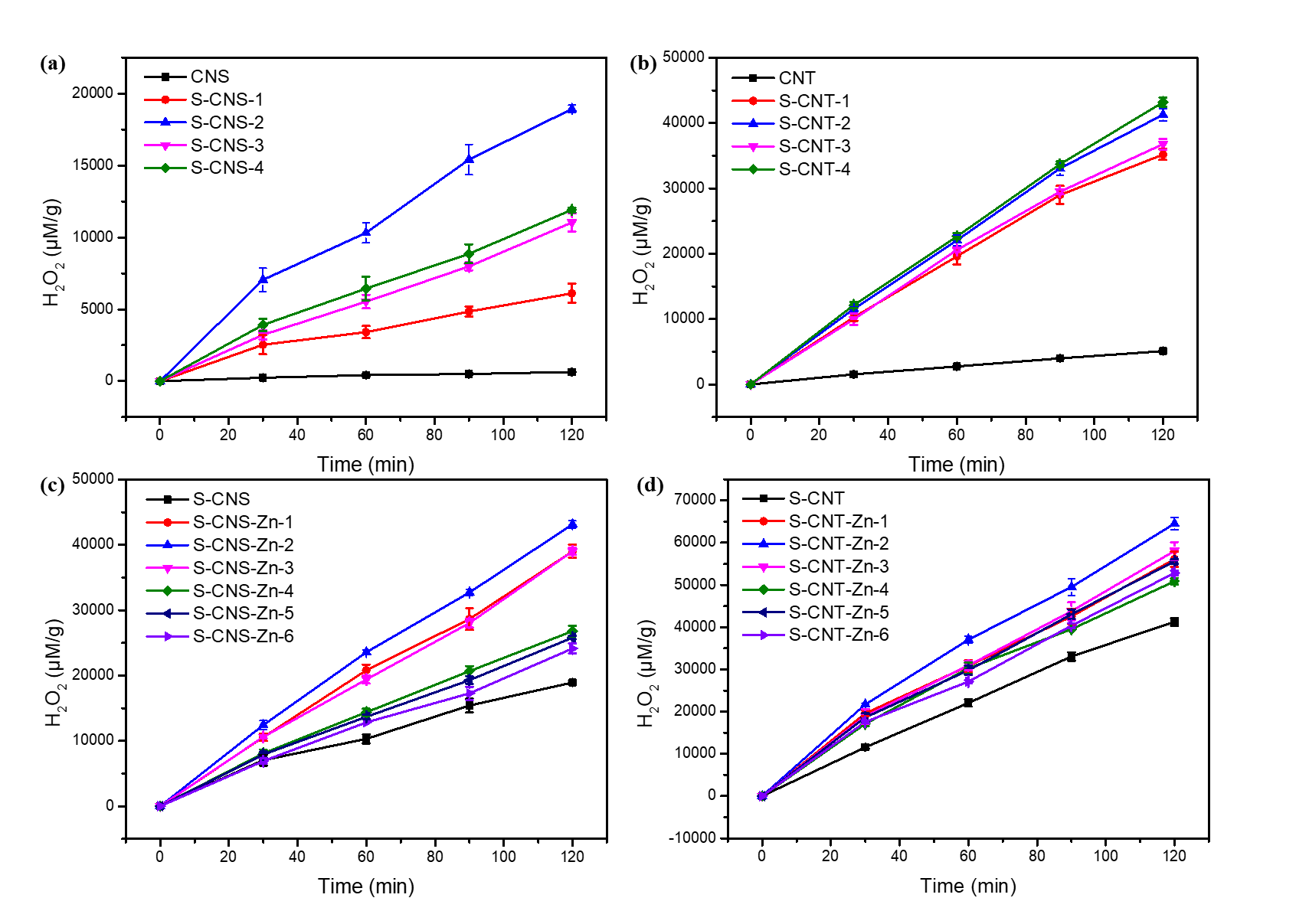
**

**Figure S1**. Photocatalytic H_2_O_2_ production activity of (a) CNS, S-CNS-1, S-CNS-2, S-CNS-3 and S-CNS-4, (b) CNT, S-CNT-1, S-CNT-2, S-CNT-3 and S-CNT-4, (c) S-CNS, S-CNS-Zn-1, S-CNS-Zn-2, S-CNS-Zn-3, S-CNS-Zn-4, S-CNS-Zn-5 and S-CNS-Zn-6, (d) S-CNT, S-CNT-Zn-1, S-CNT-Zn-2, S-CNT-Zn-3, S-CNT-Zn-4, S-CNT-Zn-5 and S-CNT-Zn-6, respectively.

**
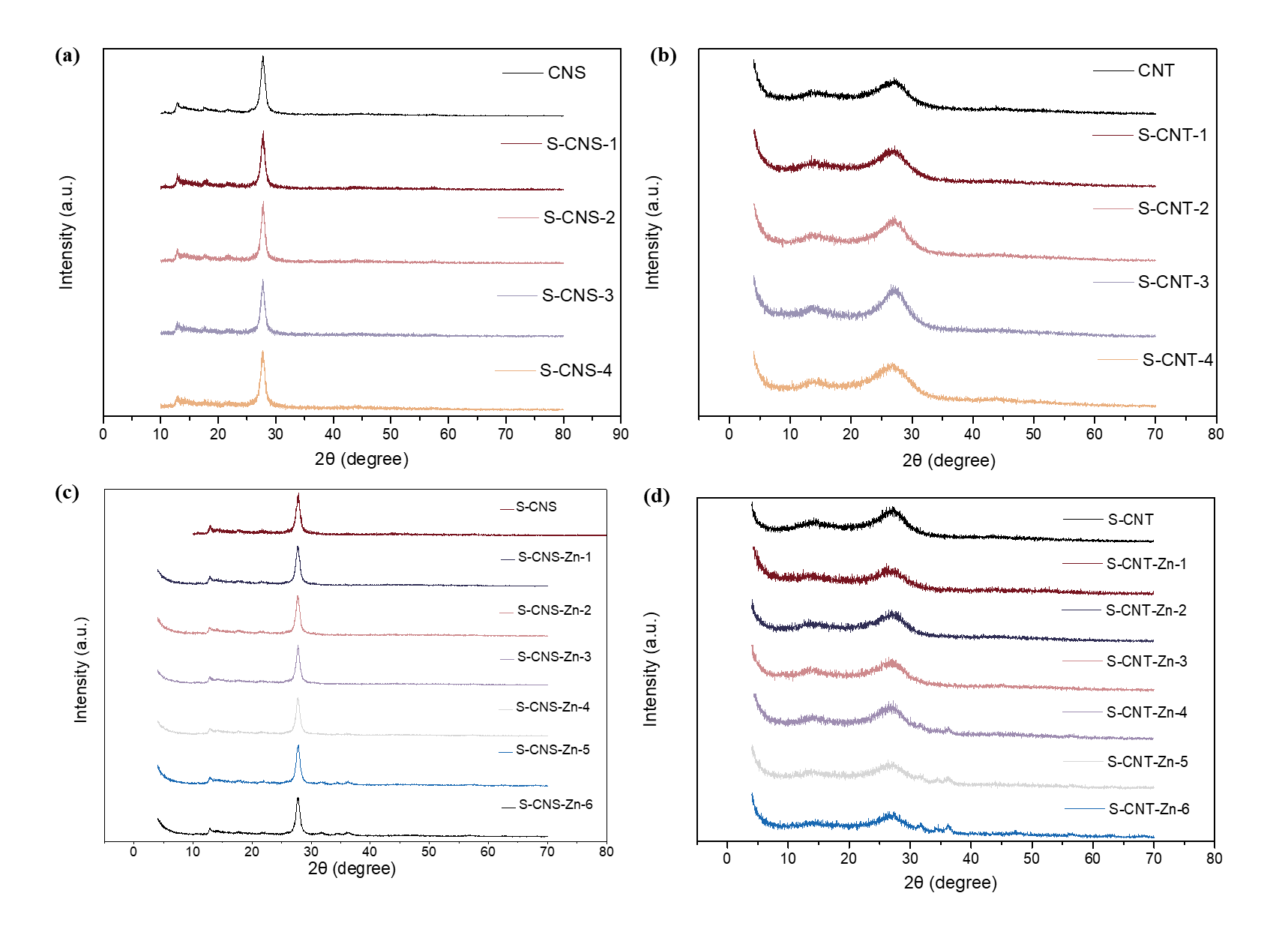
**

**Figure S2**. XRD patterns of (a) CNS, S-CNS-1, S-CNS-2, S-CNS-3 and S-CNS-4, (b) CNT, S-CNT-1, S-CNT-2, S-CNT-3 and S-CNT-4, (c) S-CNS, S-CNS-Zn-1, S-CNS-Zn-2, S-CNS-Zn-3, S-CNS-Zn-4, S-CNS-Zn-5 and S-CNS-Zn-6, (d) S-CNT, S-CNT-Zn-1, S-CNT-Zn-2, S-CNT-Zn-3, S-CNT-Zn-4, S-CNT-Zn-5 and S-CNT-Zn-6, respectively.


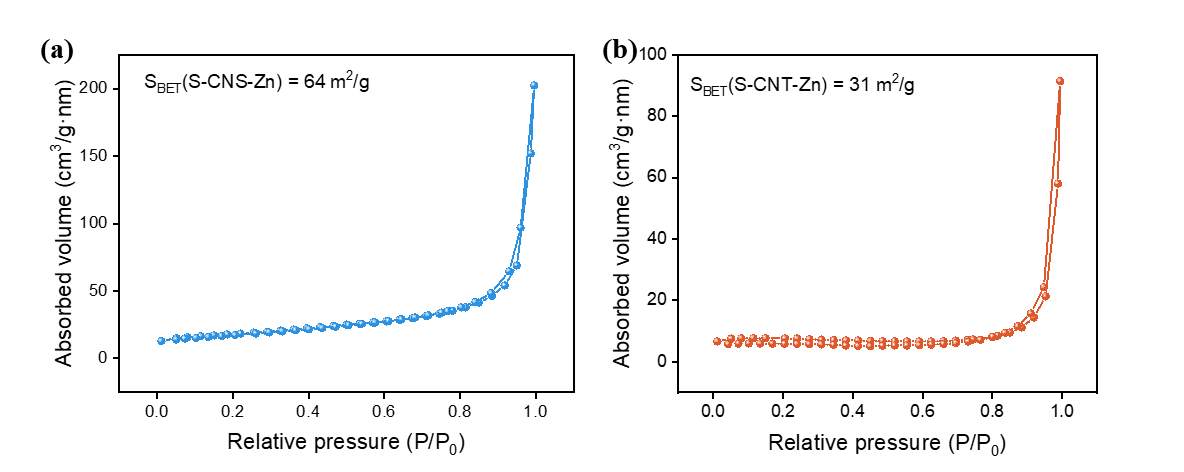


**Figure S3**. N_2_ adsorption isotherms of (a) S-CNS-Zn and (b) S-CNT-Zn.


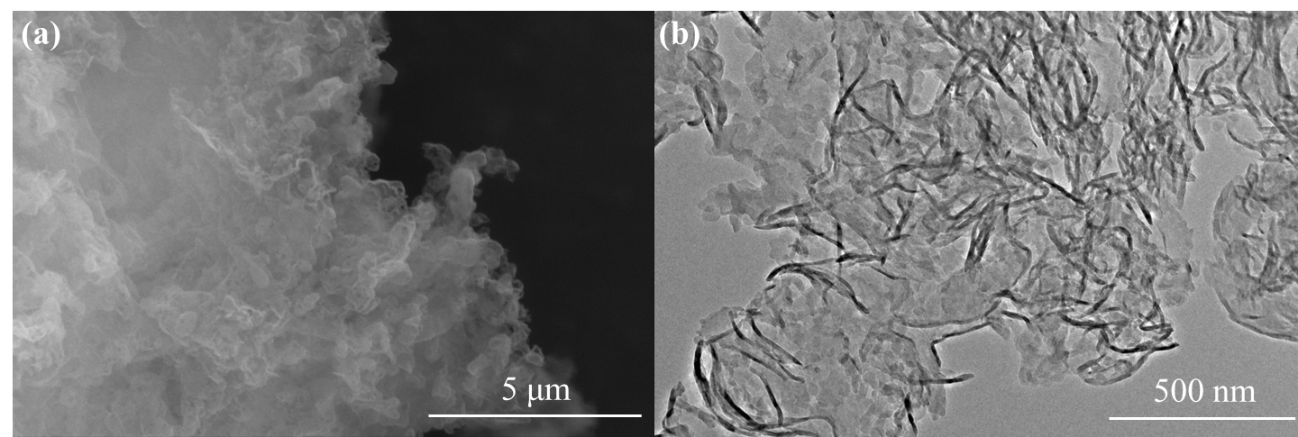


**Figure S4**. (a) SEM and (b) TEM images of CNS.


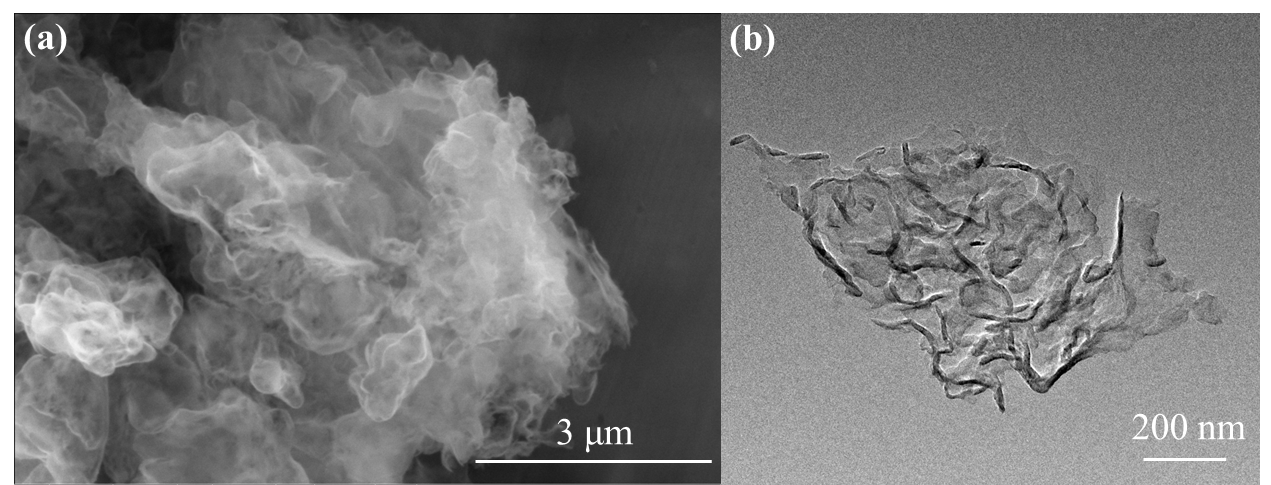


**Figure S5**. (a) SEM and (b) TEM images of S-CNS.


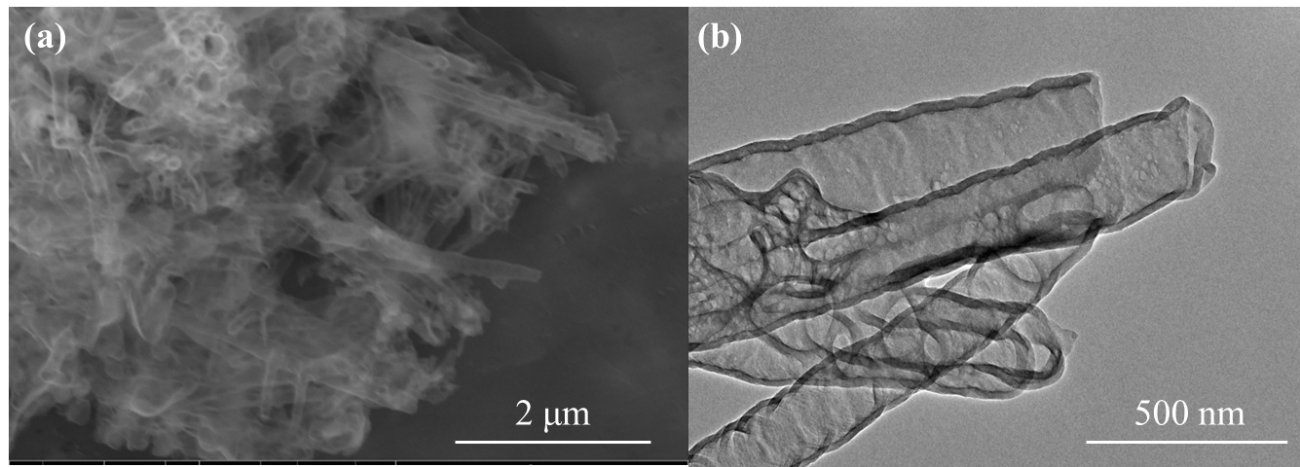


**Figure S6**. (a) SEM and (b) TEM images of CNT.


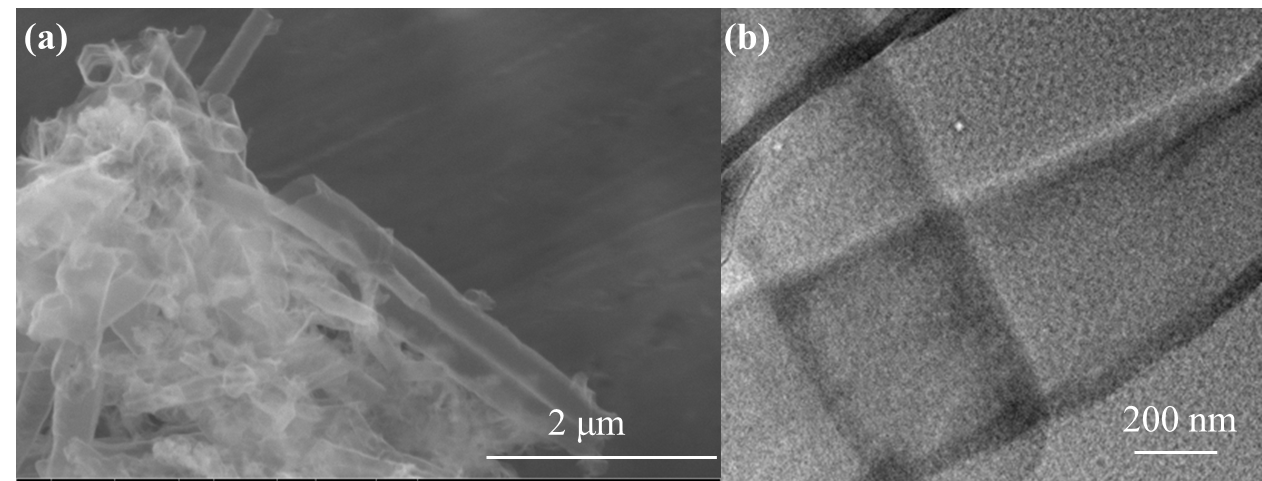


**Figure S7**. (a) SEM and (b) TEM images of S-CNT.


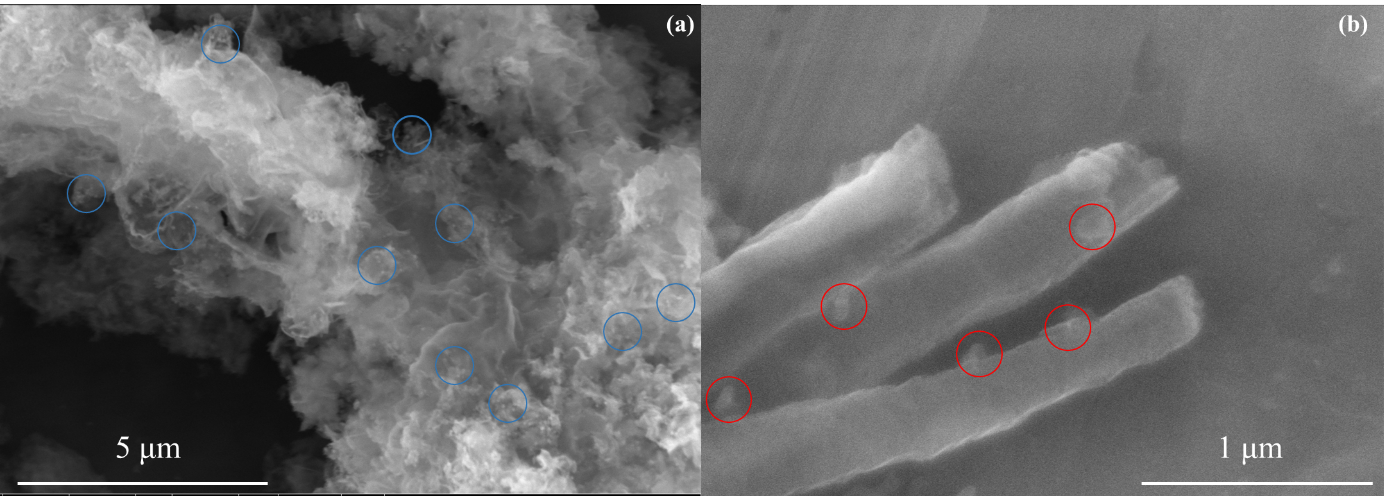


**Figure S8**. SEM images of (a) S-CNS-Zn-4 and (b) S-CNT-Zn-4, in which the circles indicate the ZnO particles.


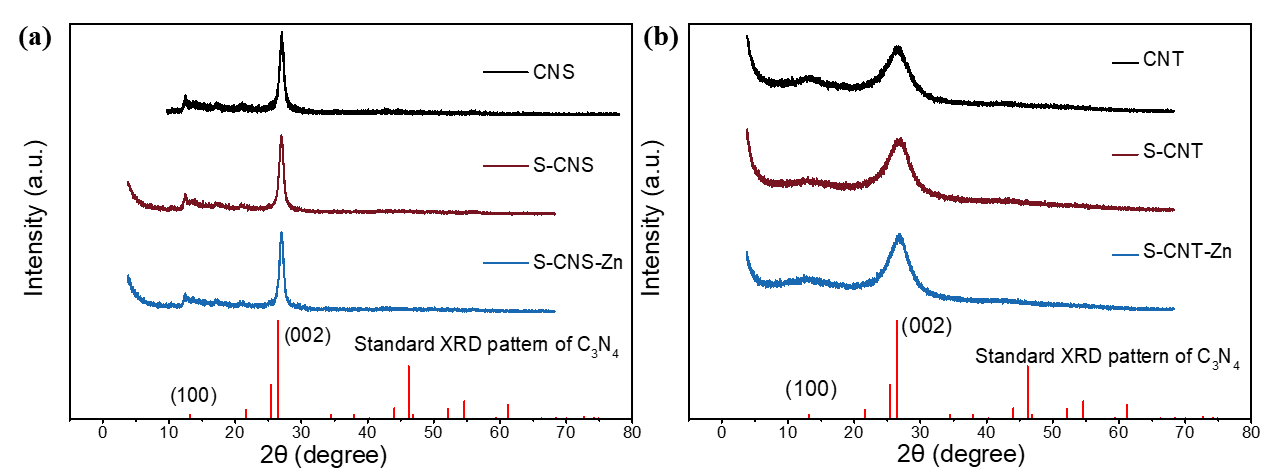


**Figure S9**. XRD patterns of (a) CNS, S-CNS and S-CNS-Zn and (b) CNT, S-CNT and S-CNT-Zn.


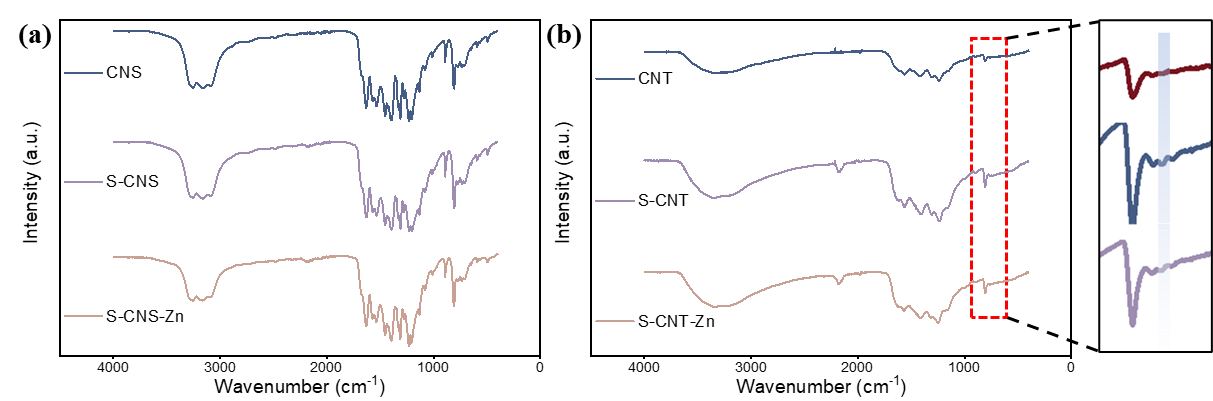


**Figure S10**. FTIR spectra of (a) CNS, S-CNS and S-CNS-Zn and (b) CNT, S-CNT and S-CNT-Zn.


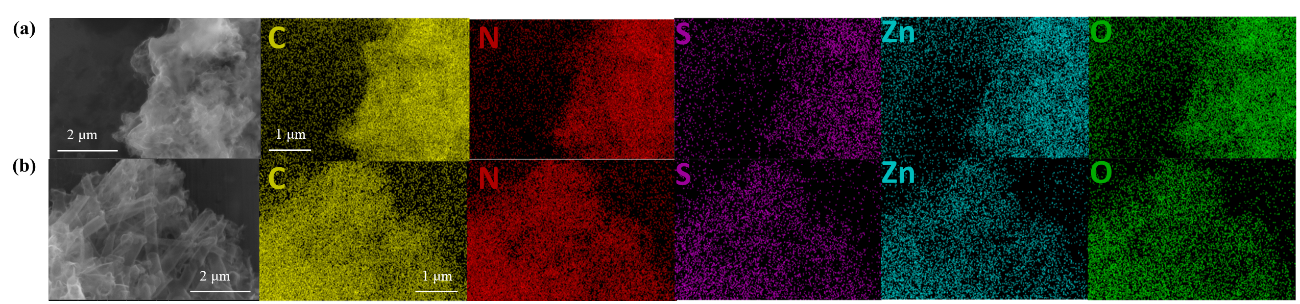


**Figure. S11**. EDS mapping images of (a) S-CNS-Zn and (b) S-CNT-Zn.


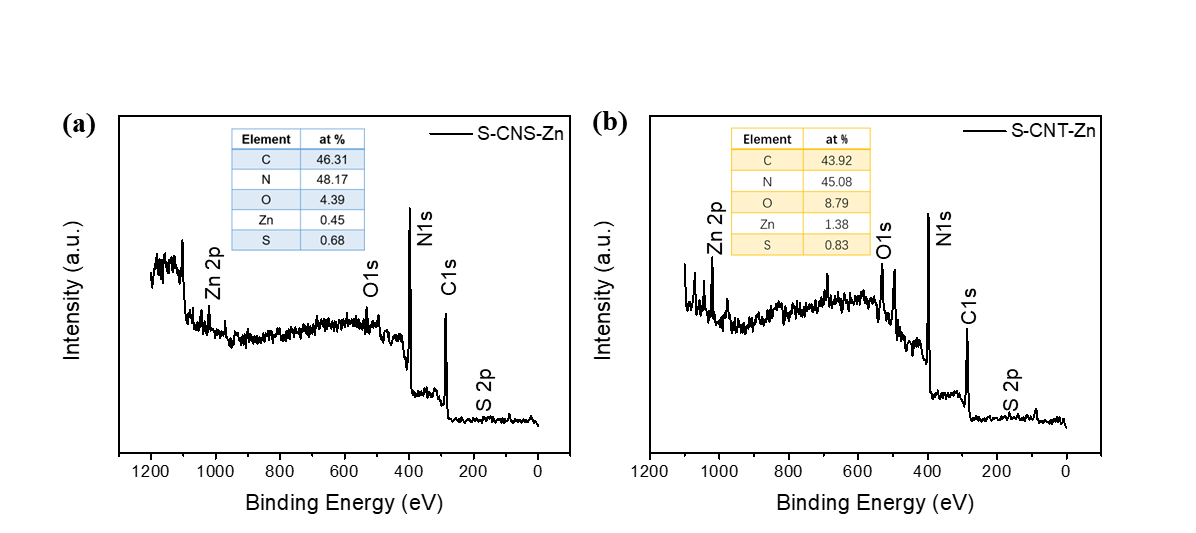


**Figure S12**. XPS survey spectra and corresponding element compositions of (a) S-CNS-Zn and (b) S-CNT-Zn.


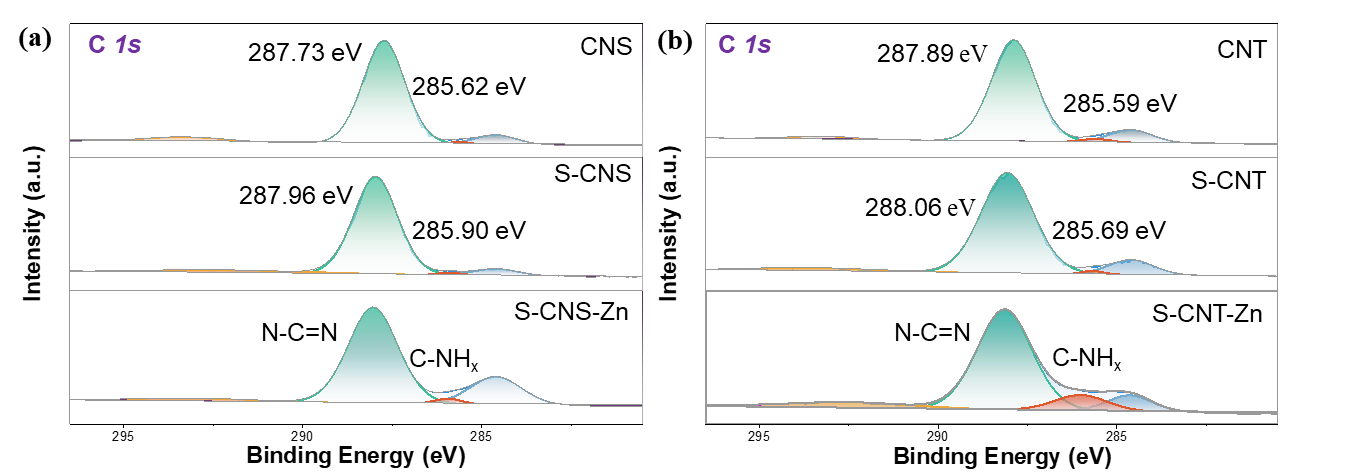


**Figure S13**. High-resolution C 1s XPS spectra of (a) S-CNS-Zn and (b) S-CNT-Zn.


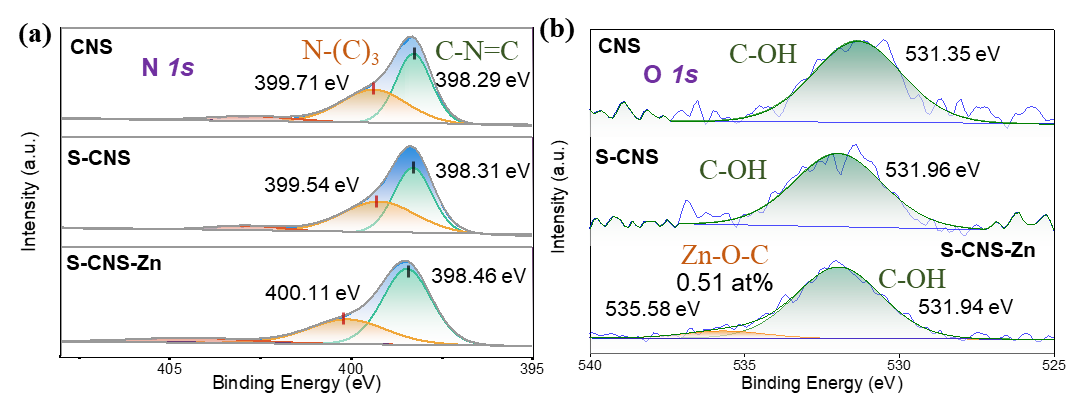


**Figure S14**. High-resolution (a) N 1s and (b) O 1s XPS spectra of CNS, S-CNS, and S-CNS-Zn, respectively.


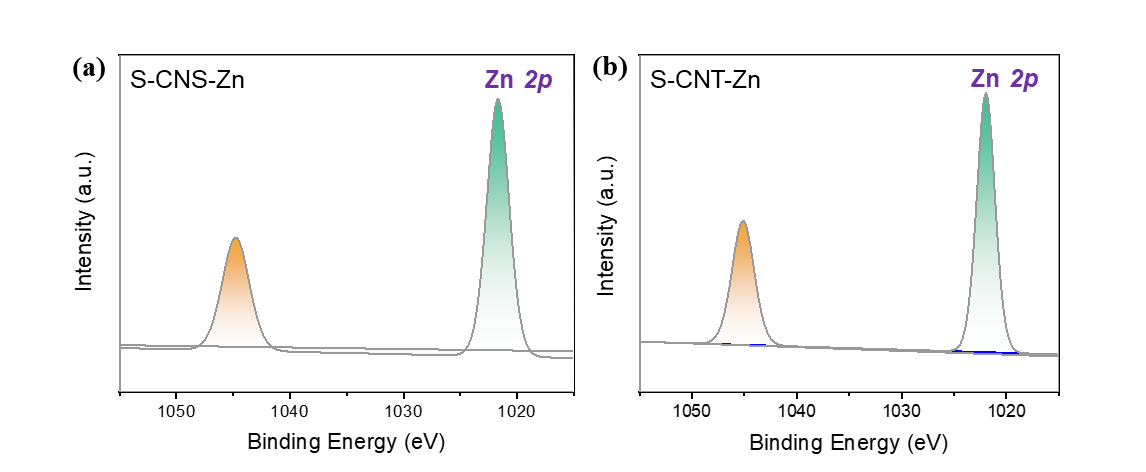


**Figure S15**. High-resolution Zn 2p XPS spectra of (a) S-CNS-Zn and (b) S-CNT-Zn.


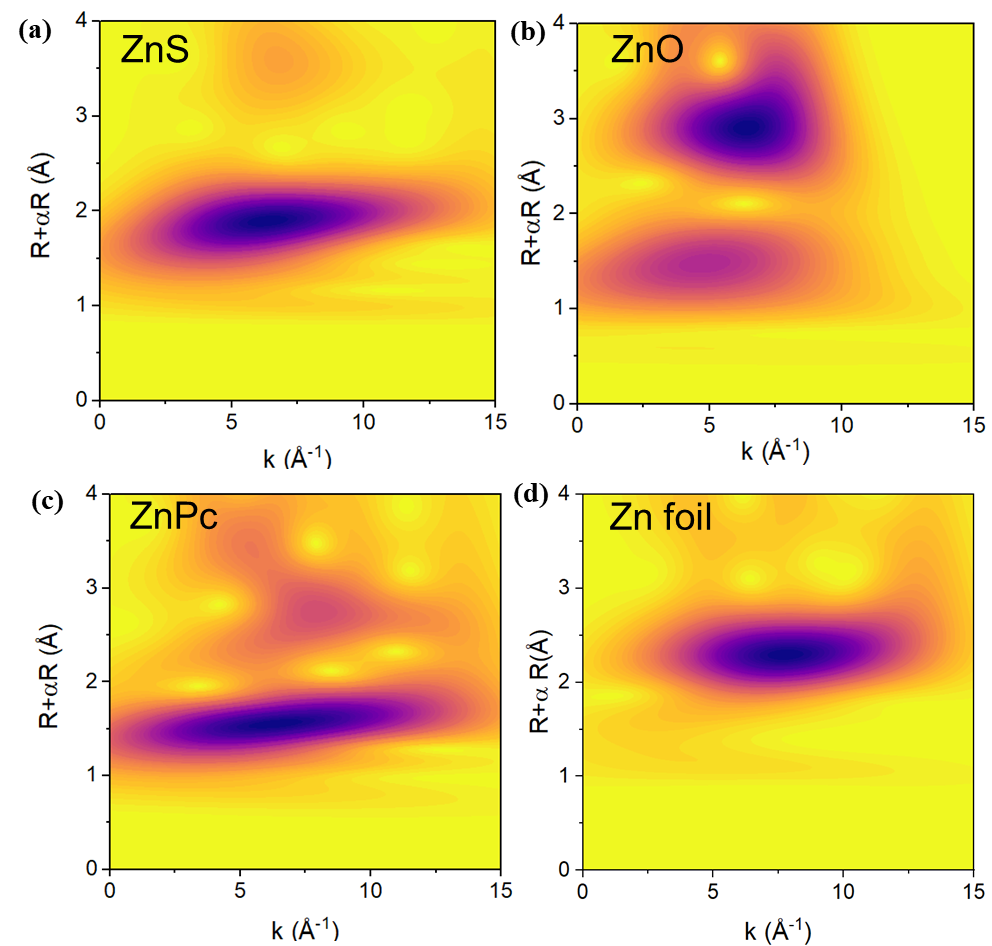


**Figure S16**. Wavelet transforms of the EXAFS signals of (a) ZnS, (b) ZnO, (c) ZnPc, and (d) Zn foil.


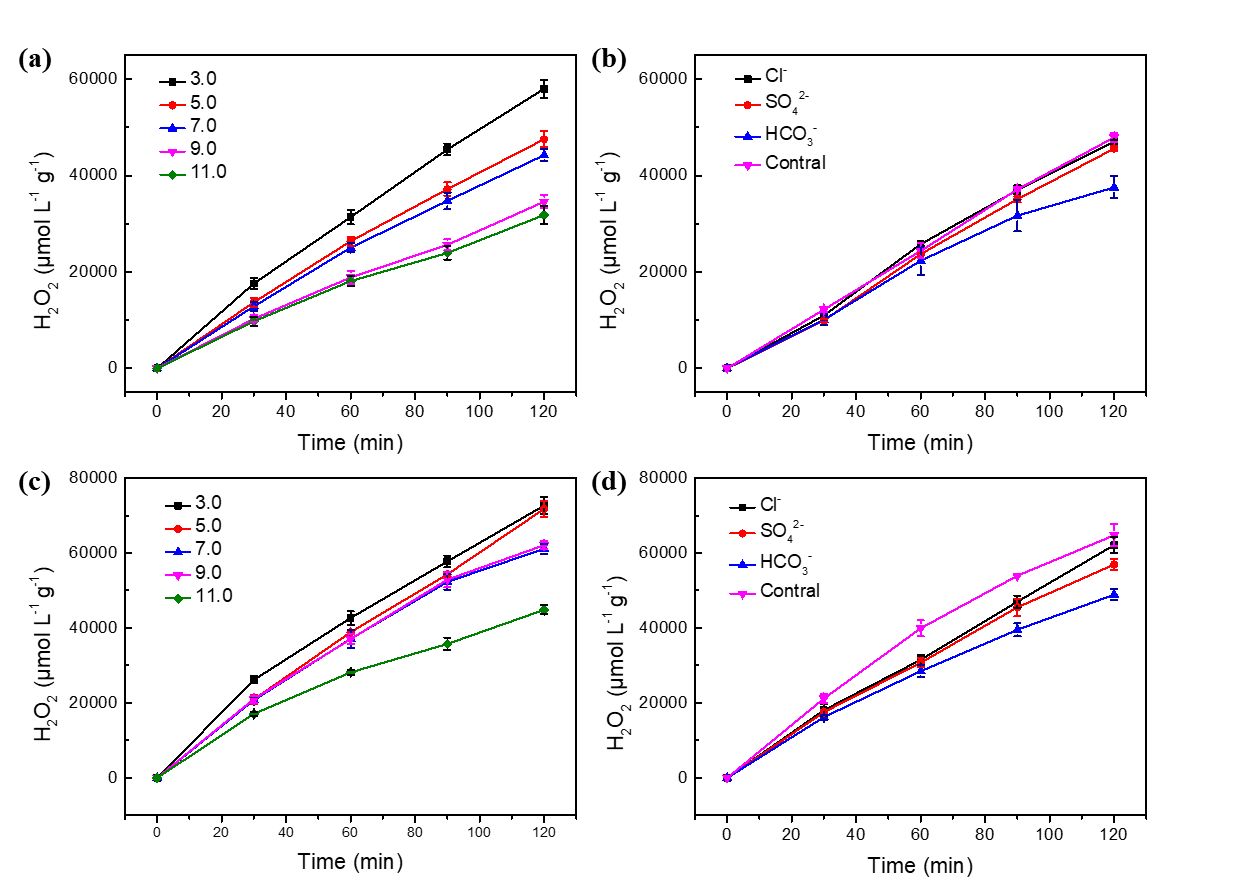


**Figure S17**. Activity comparison of (a, b) S-CNS-Zn and (c, d) S-CNT-Zn in the different pH conditions and presence of 0.2 mmol L^–1^ NaCl, NaSO_4_ and NaHCO_3_, respectively.


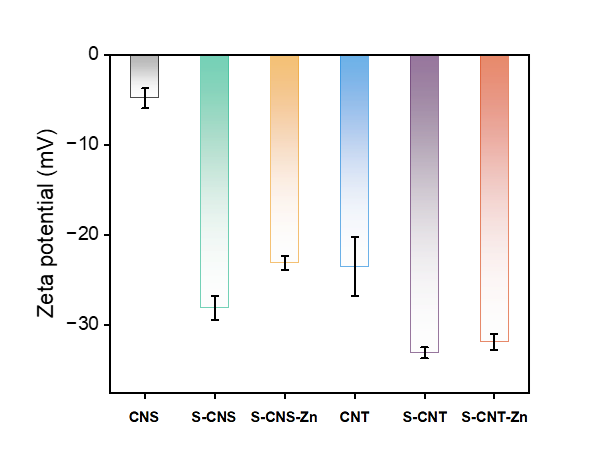


**Figure S18**. Zeta potentials of CNS, S-CNS, S-CNS-Zn, CNT, S-CNT

and S-CNT-Zn.


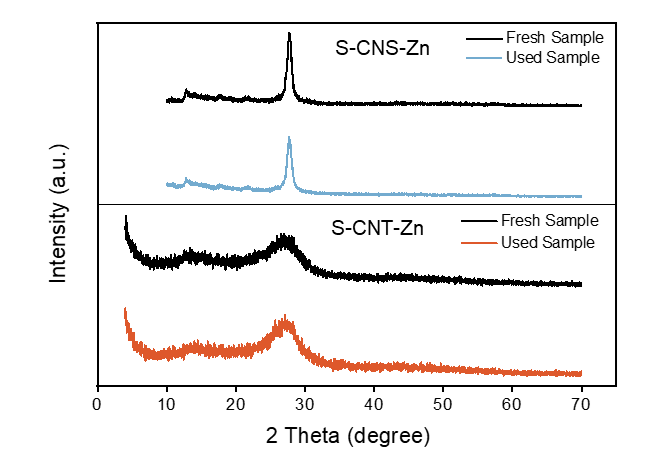


**Figure S19**. XRD patterns of fresh and used S-CNS-Zn and S-CNT-Zn.


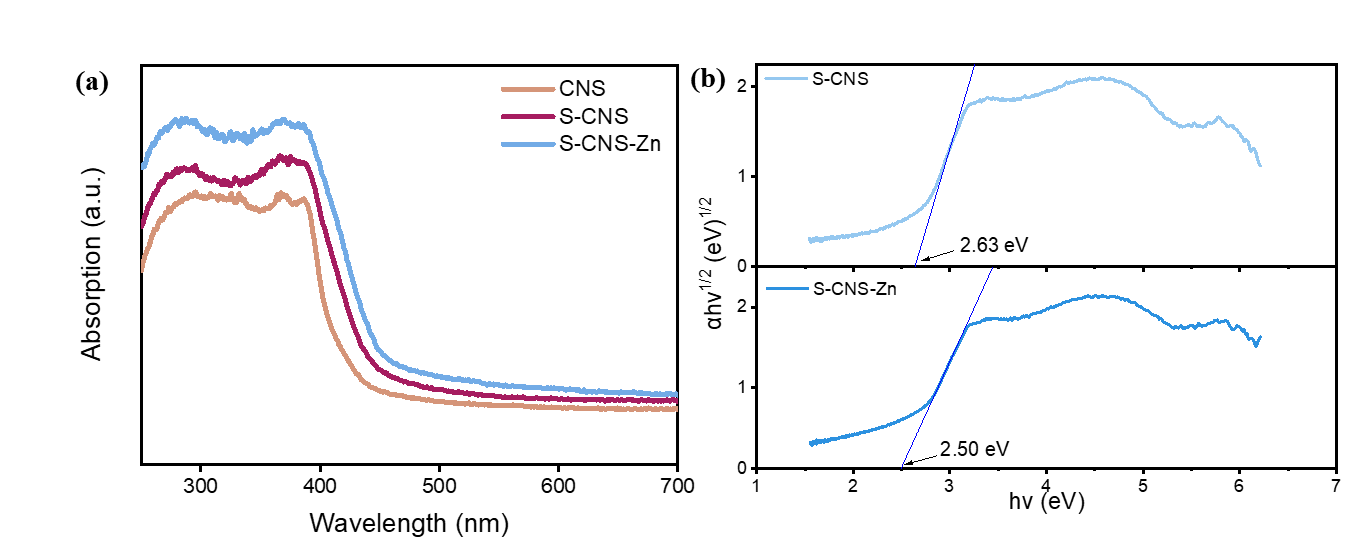


**Figure S20**. (a) UV/Vis diffuse reflectance spectra and (b) the bandgaps of S-CNS and S-CNS-Zn.


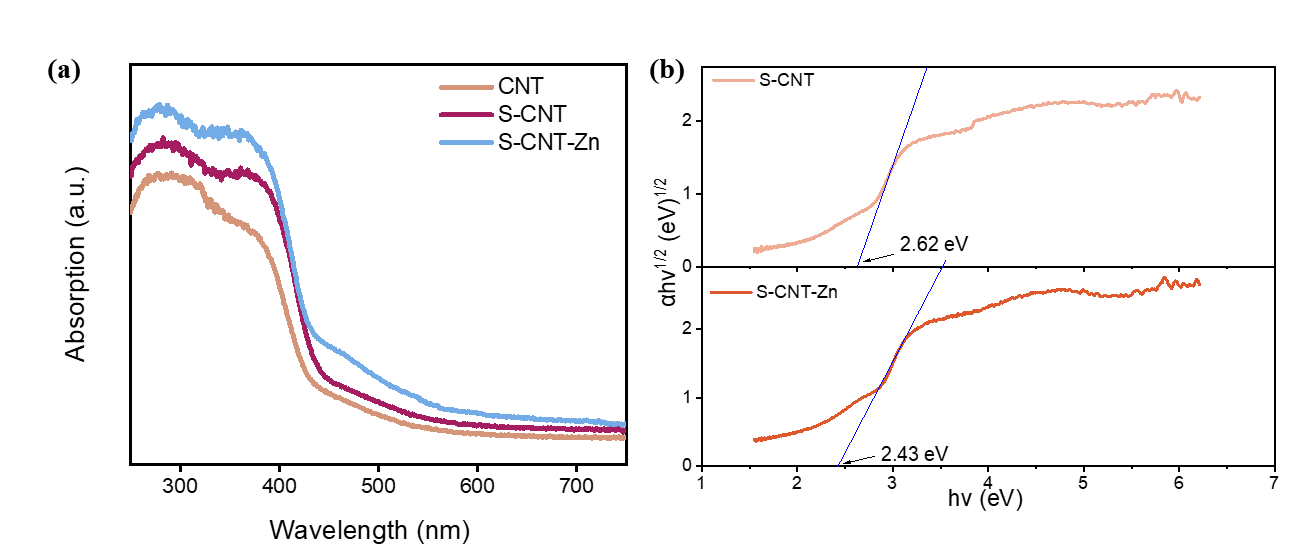


**Figure S21**. (a) UV/Vis diffuse reflectance spectra and (b) the bandgaps of S-CNT and S-CNT-Zn.


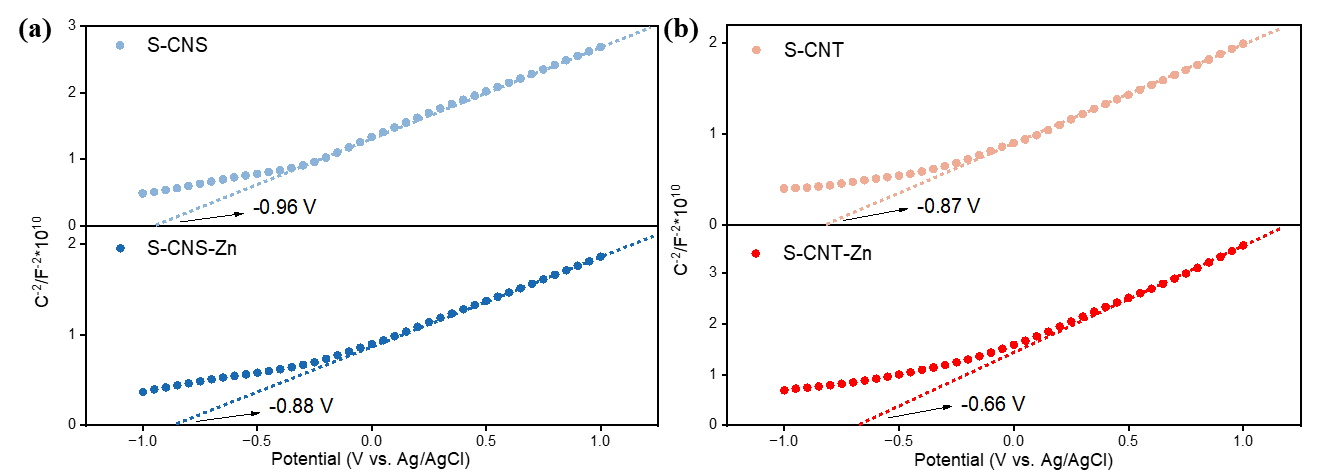


**Figure S22**. Mott-Schottky plots of (a) S-CNS and S-CNS-Zn and (b) S-CNT and S-CNT-Zn, respectively.


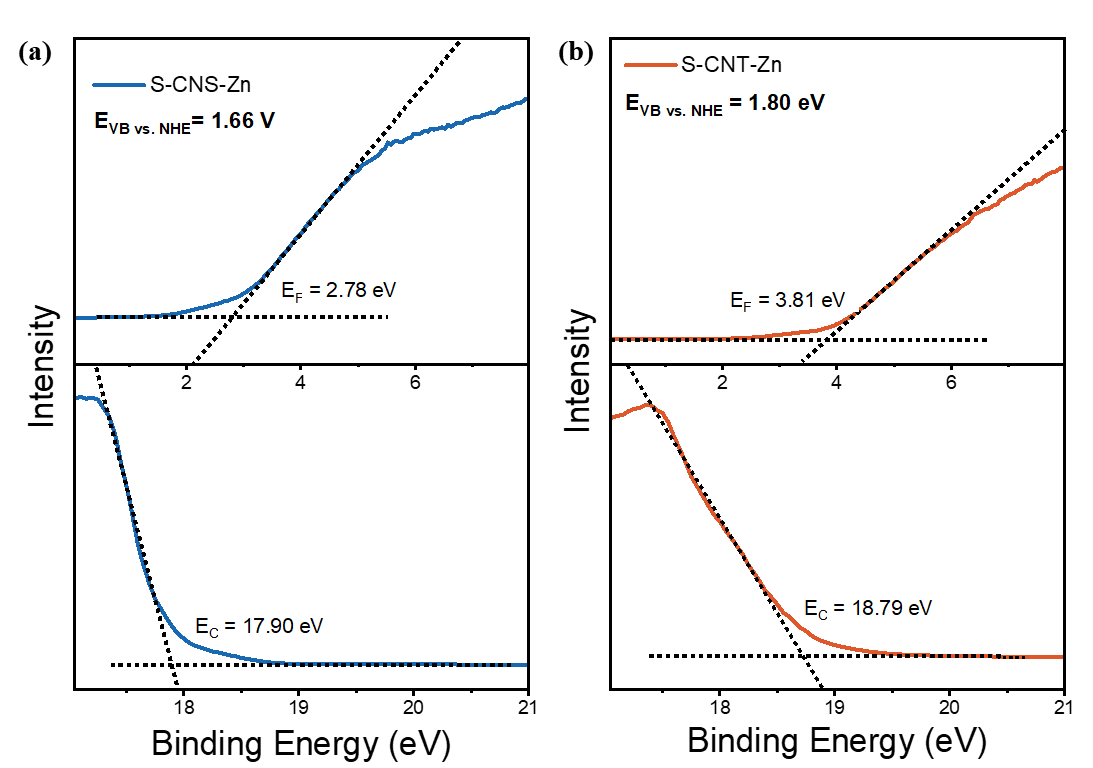


**Figure S23.** Fermi levels and high kinetic energy region of UPS spectra from (a) S-CNS-Zn and (b) S-CNT-Zn, respectively.

**
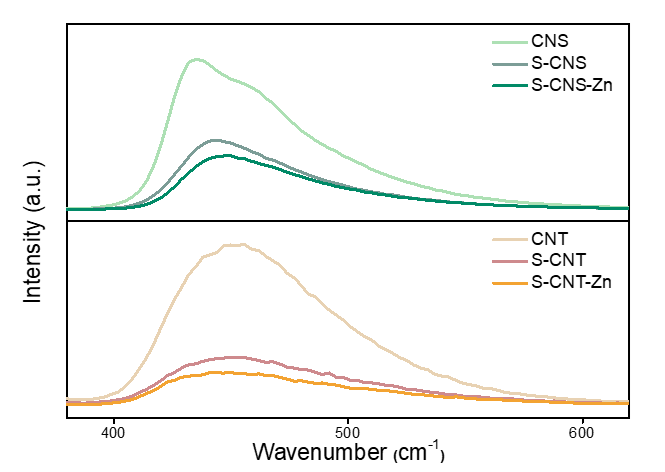
**

**Figure S24**. PL spectra of CNS, S-CNS, S-CNS-Zn, CNT, S-CNT and S-CNT-Zn.


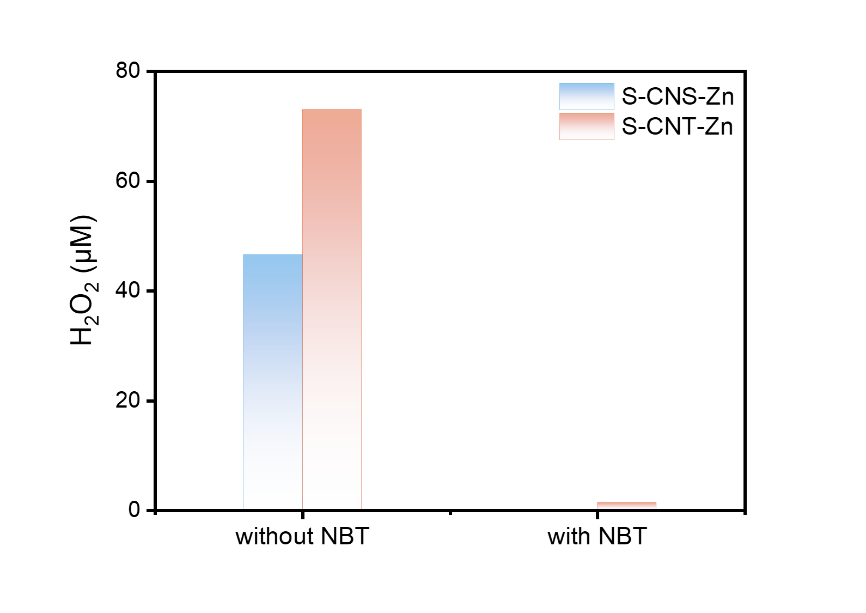


**Figure S25**. Activity comparison of S-CNS-Zn and S-CNT-Zn with and without nitroblue tetrazolium chloride (NBT, a sacrificial agent for •O_2_^–^).


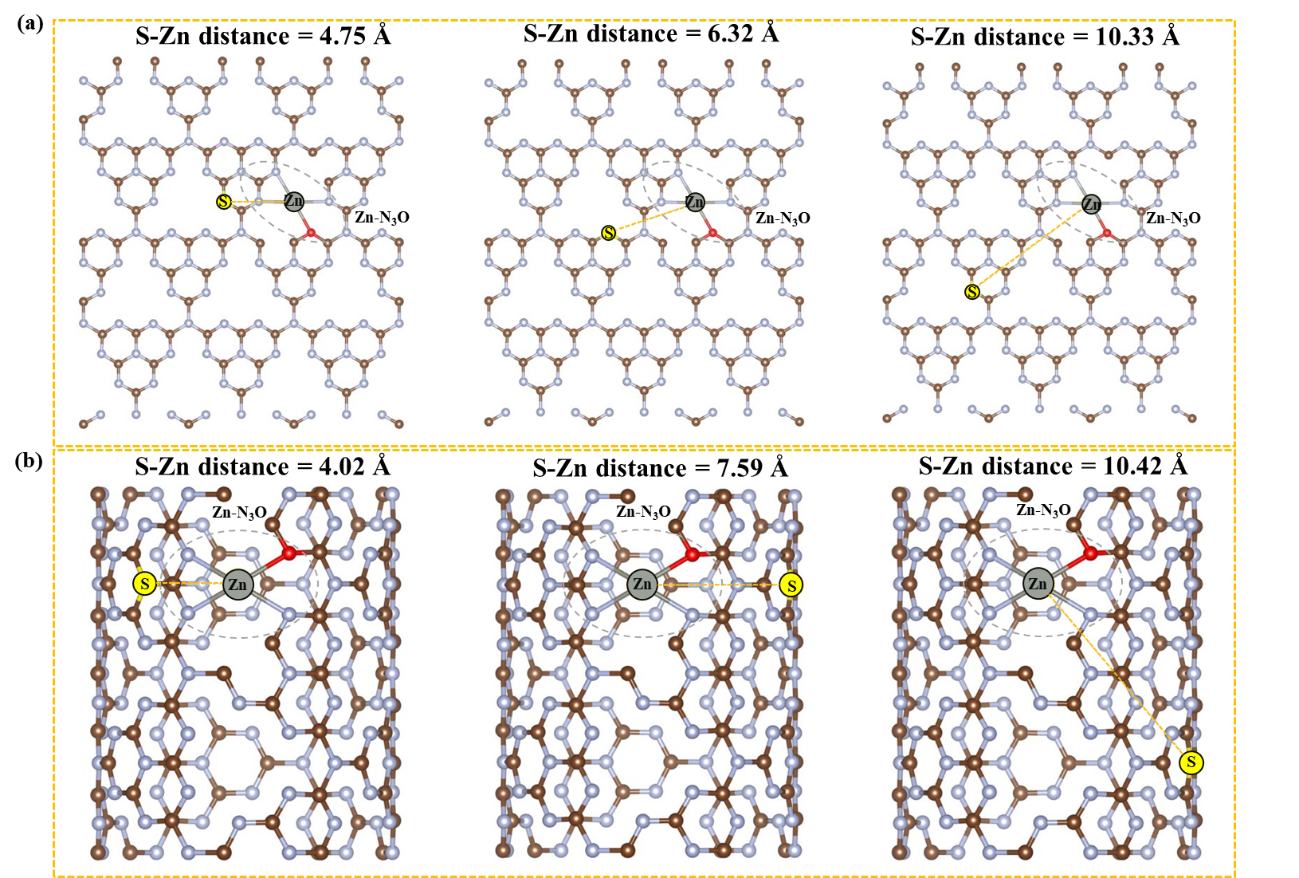


**Figure S26**. The different relative positions of S sites and Zn center of Zn-N_3_O on (a) S-CNS-Zn and (b) S-CNT-Zn.


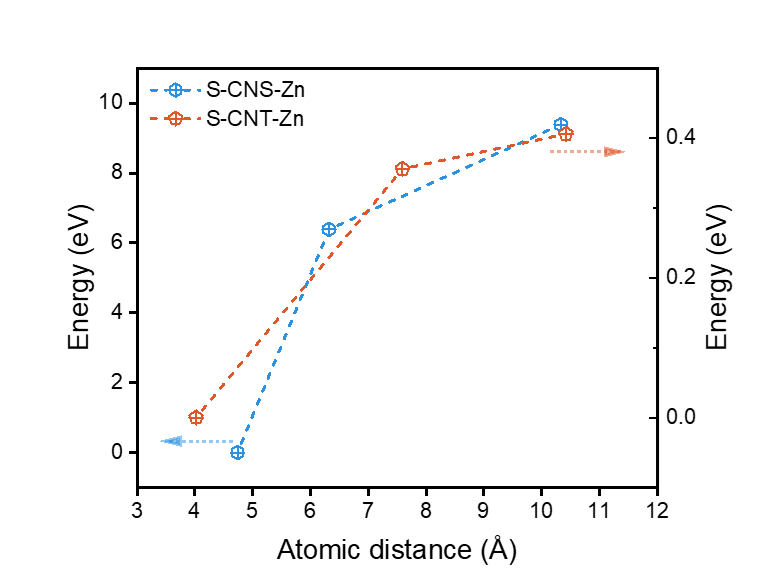


**Figure S27**. Energy at different relative distances of Zn and S sites on S-CNS-Zn and S-CNT-Zn according to Figure S26.


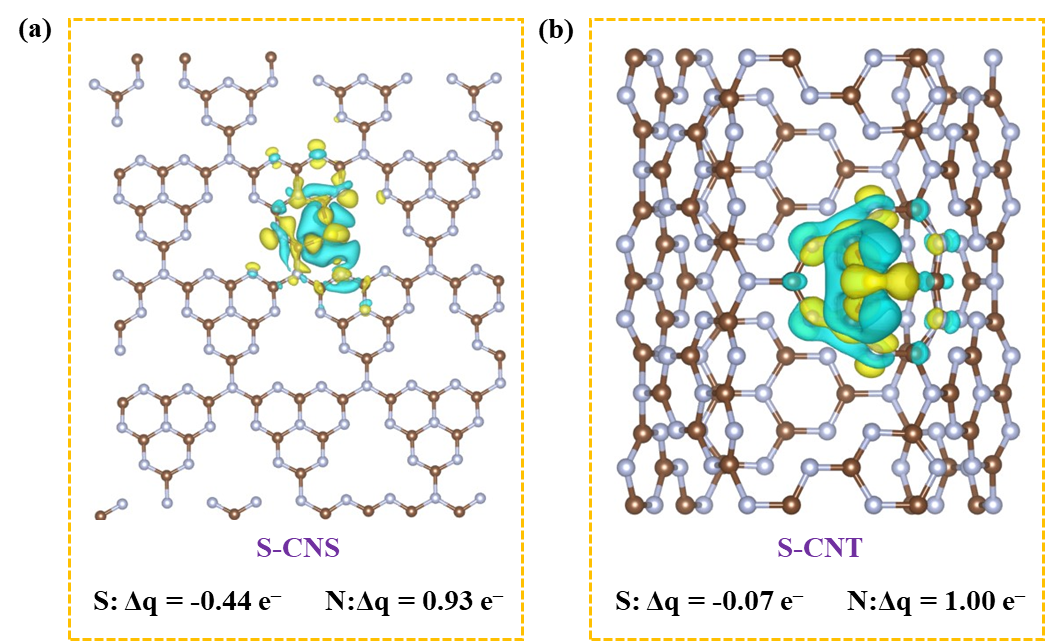


**Figure S28**. Differential charge density of (a) S-CNS and (b) S-CNT.


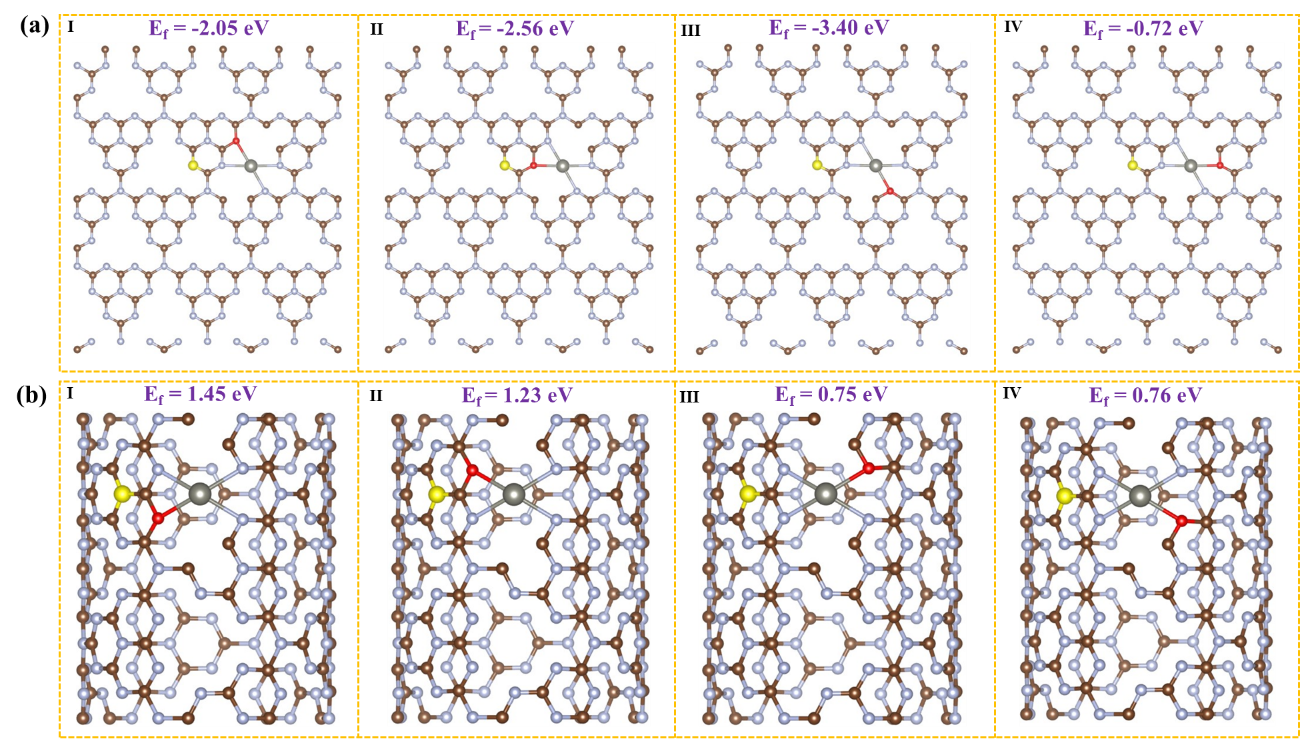


**Figure S29**. The formation energy at different relative positions of O sites on (a) S-CNS-Zn and (b) S-CNT-Zn.


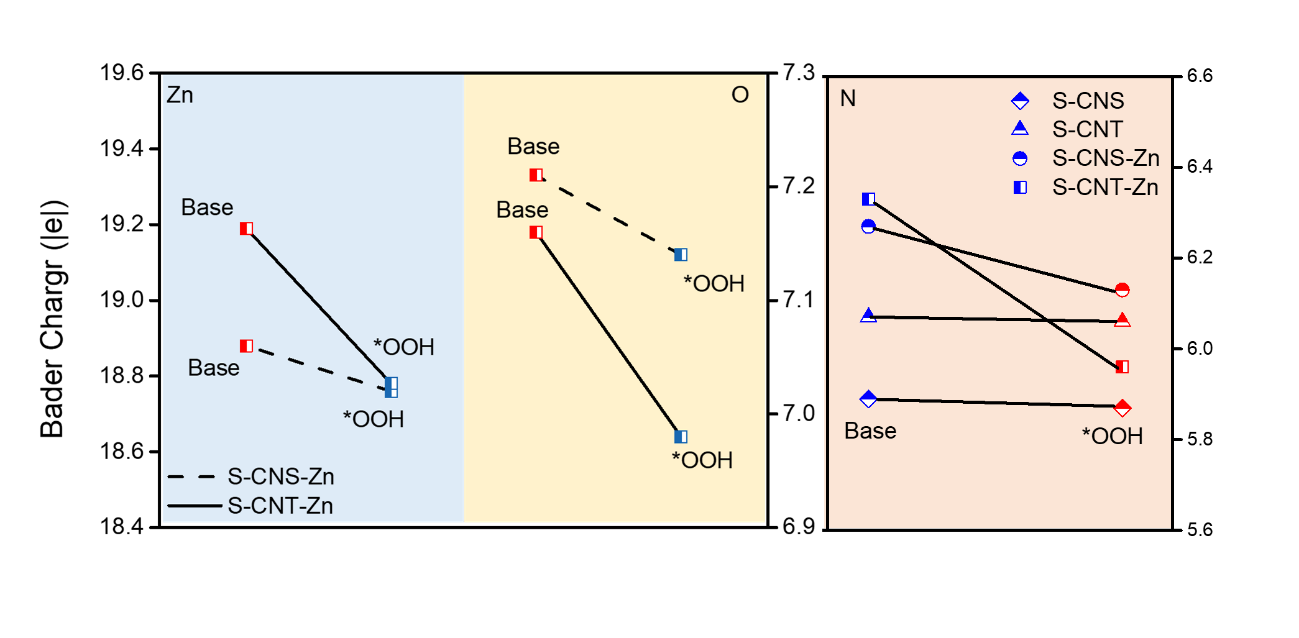


**Figure S30.** The Bader Charge of Zn atom and O atom (N atom) before and after *OOH adsorption at S-CNS, S-CNT, S-CNS-Zn and S-CNT-Zn.


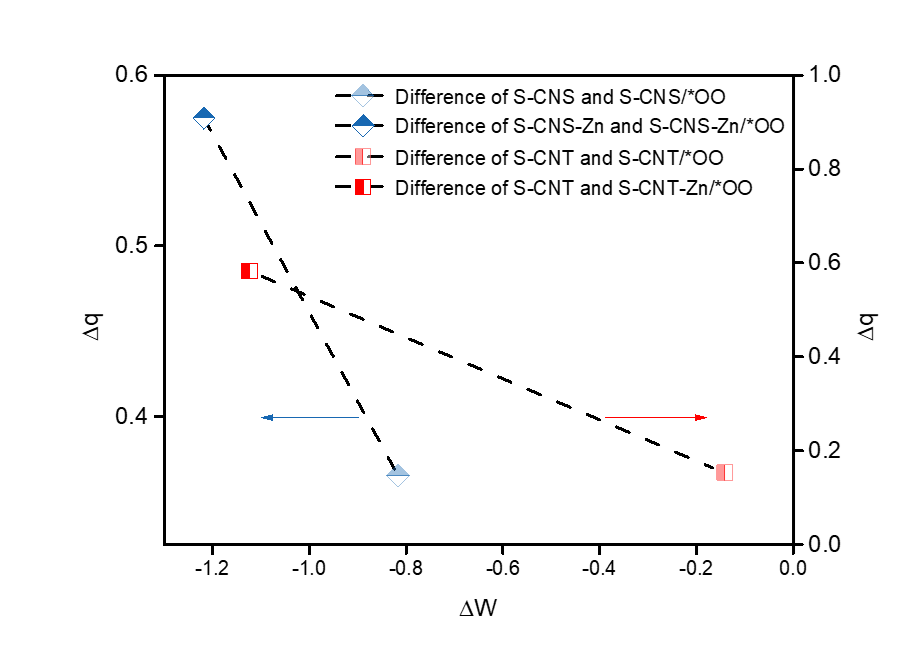


**Figure S31.** Relationship between transferred charge (Δq) and the work function difference (ΔW) between S-CNS, S-CNT, S-CNS-Zn, S-CNT-Zn and S-CNS/*OO, S-CNT/*OO, S-CNS-Zn/*OO, S-CNT-Zn/*OO, respectively.


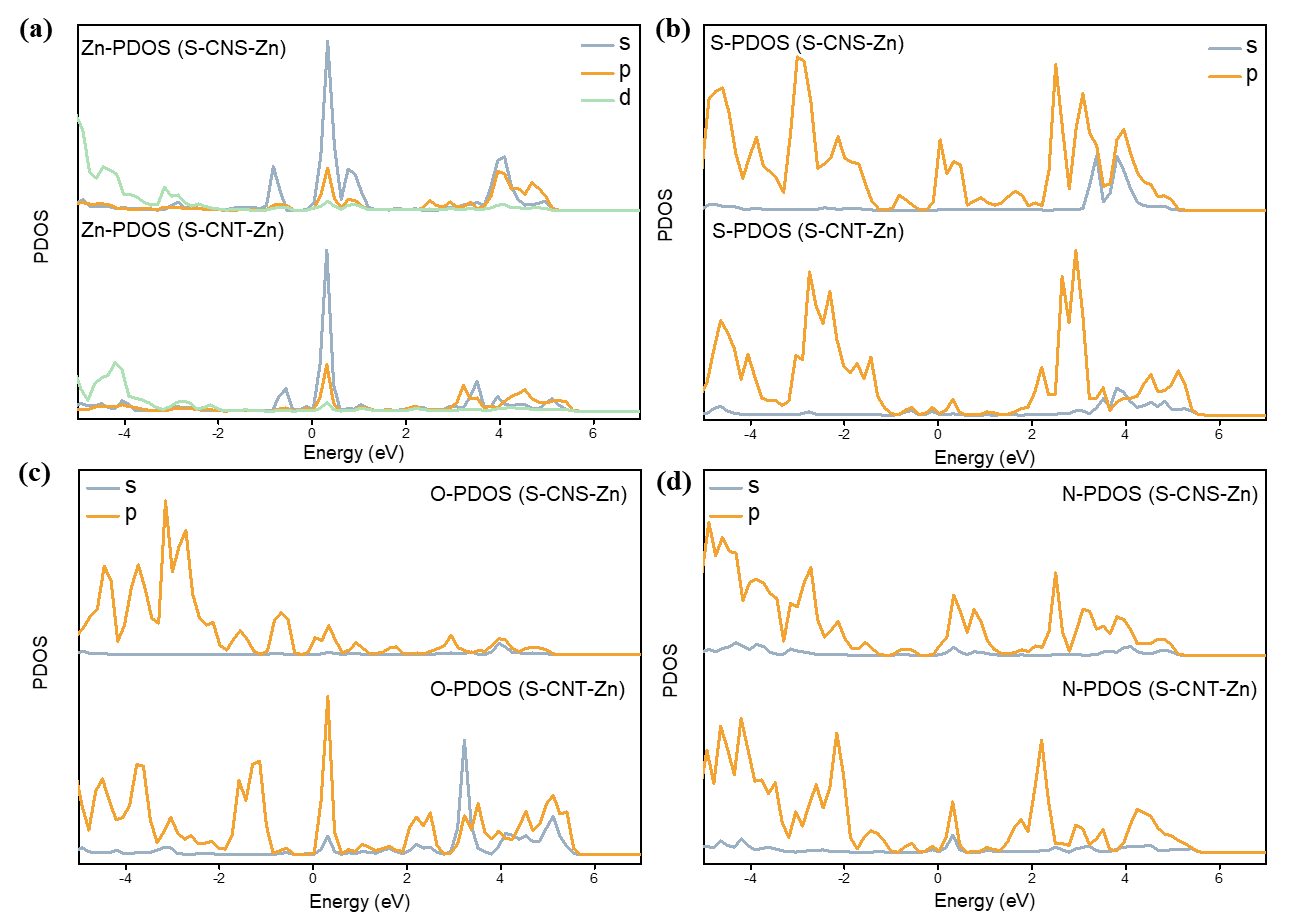


**Figure S32**. PDOS of (a) Zn, (b) S, (c) O, and (d) N in S-CNS-Zn and S-CNT-Zn, respectively.


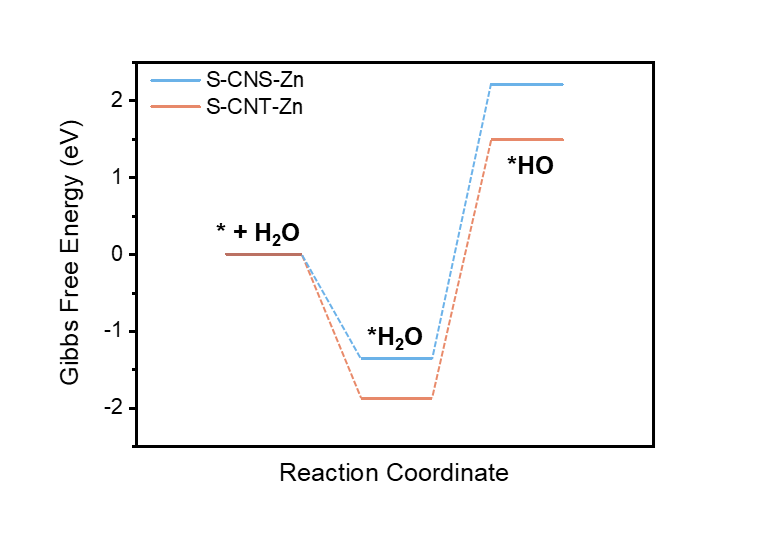


**Figure S33**. The dehydrogenation of H_2_O on the S-CNS-Zn and S-CNT-Zn.


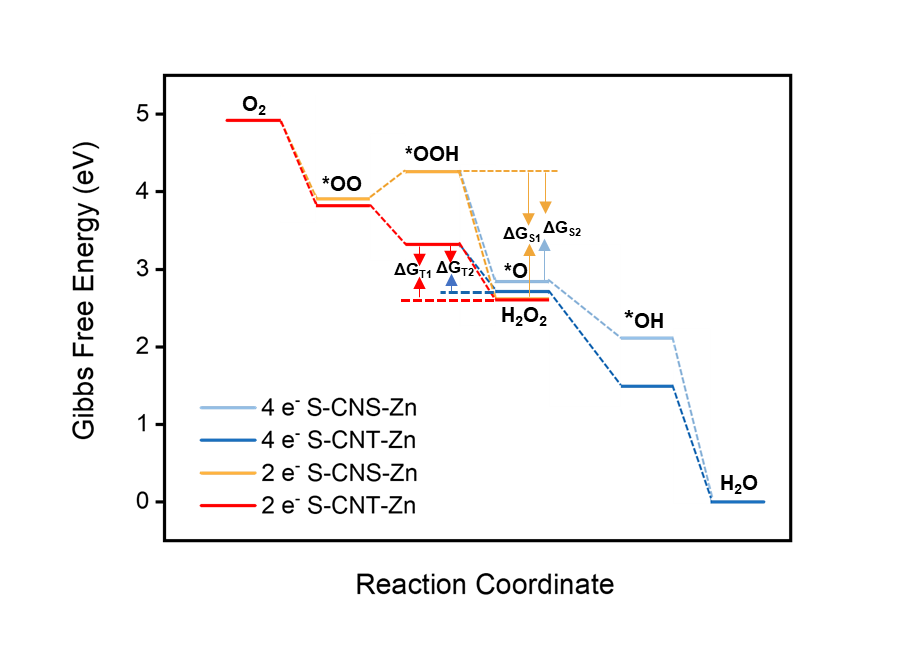


**Figure S34**. Free energy profiles of 2e^–^/4e^–^ pathway ORR for S-CNS-Zn and S-CNT-Zn, respectively.


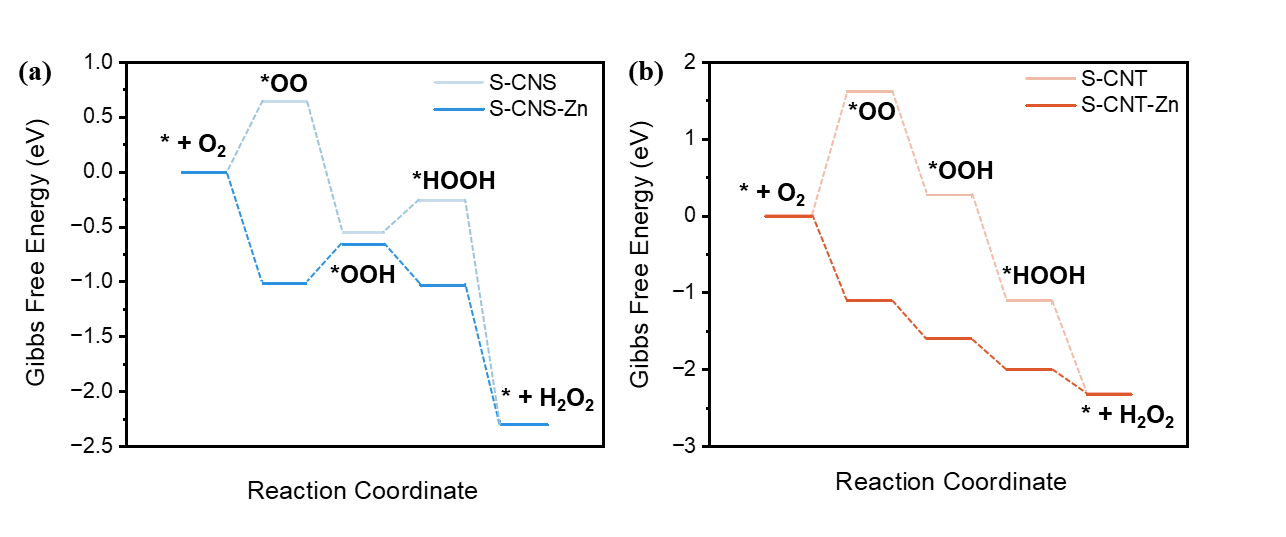


**Figure S35**. Free energy diagram of 2e^–^ O_2_ reduction with different catalysts.


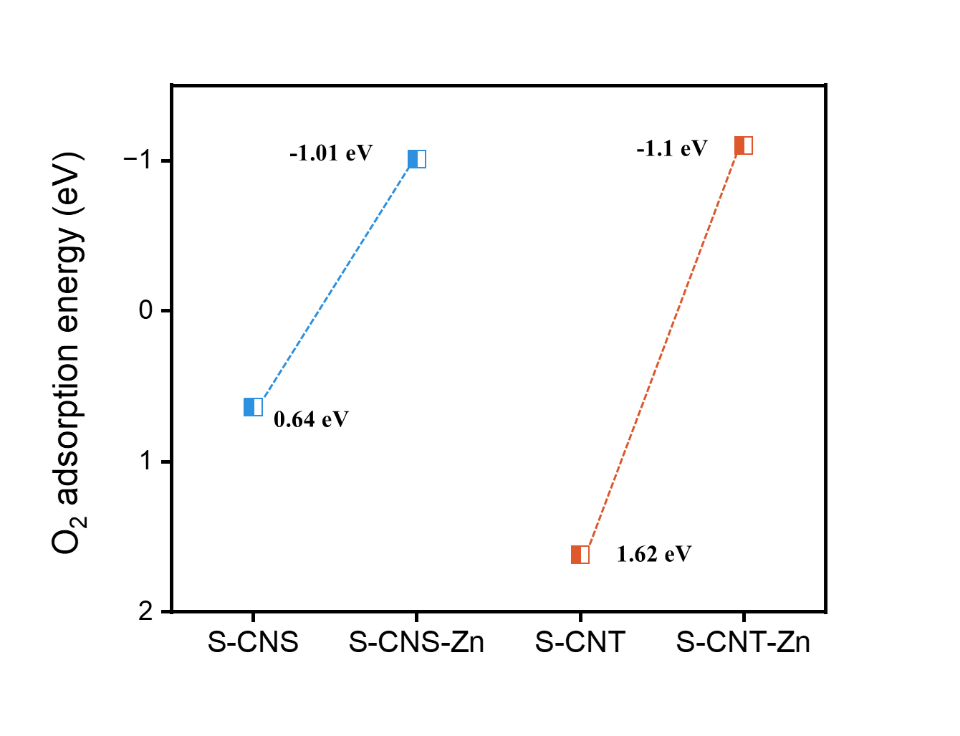


**Figure S36**. The adsorption energy of O_2_ on optimum site of S-CNS, S-CNS-Zn,

S-CNT and S-CNT-Zn.


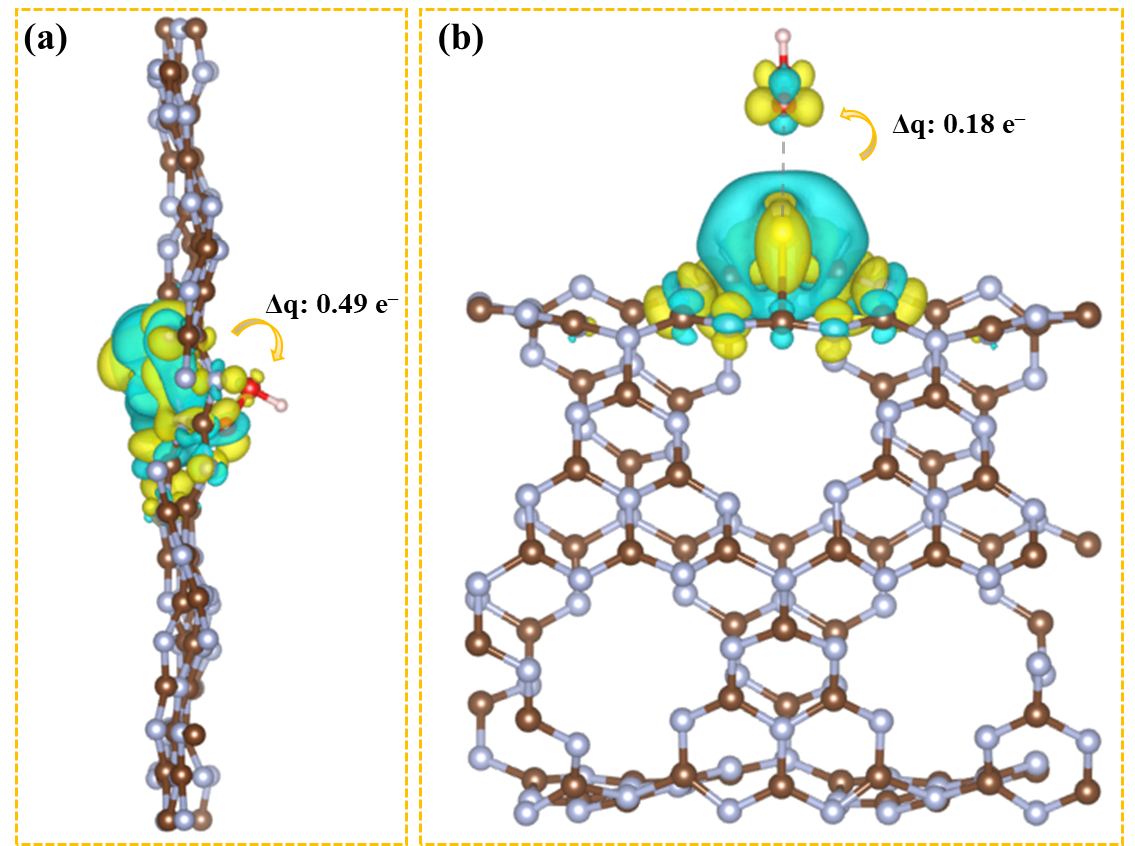


**Figure S37**. (a) S-CNS/OOH and (b) S-CNT/OOH models and the corresponding charge accumulated in *OOH intermediate.


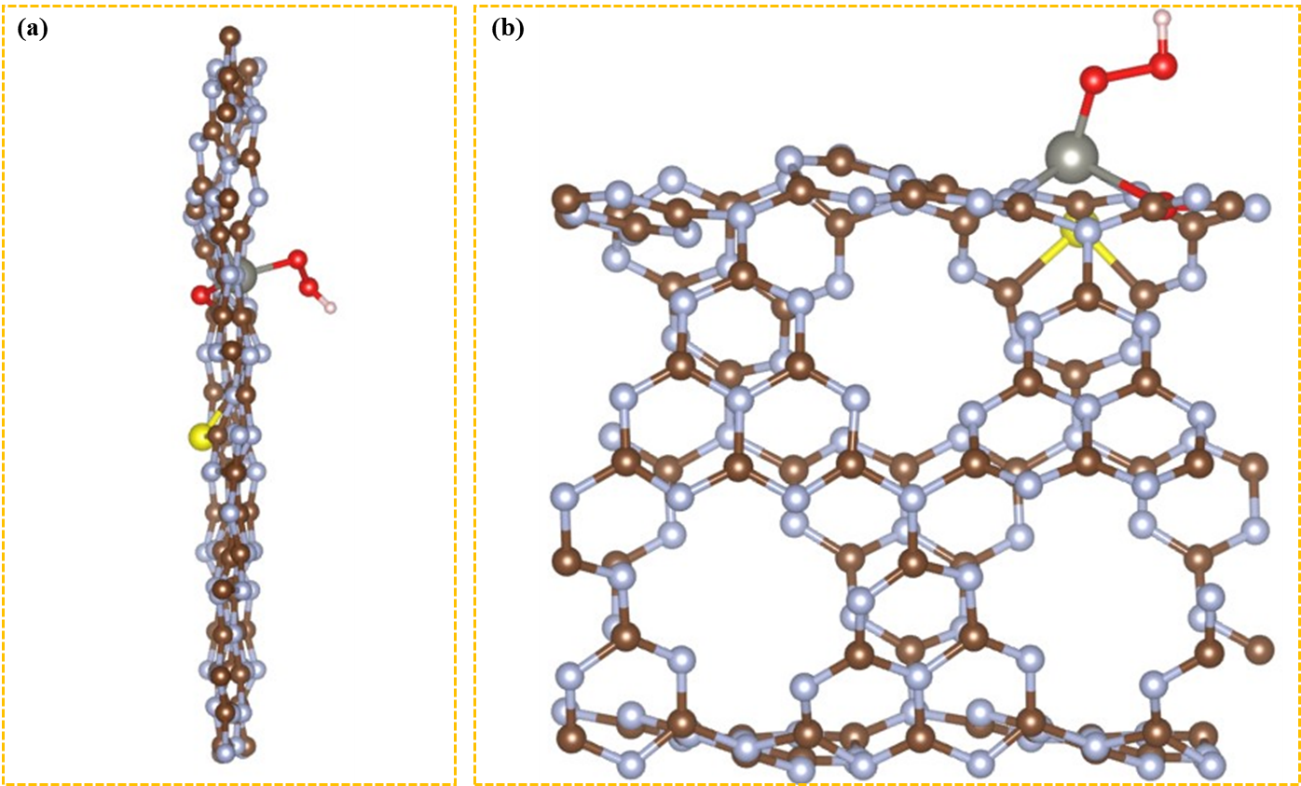


**Figure S38**. Calculation model of (a) S-CNS-Zn/OOH and (b) S-CNT-Zn/OOH from the side view.

**Table S1**. N_2C_/N_3C_ atomic ratios of samples determined by XPS

| **Sample** | **N_3C_ (%)** | **N_2C_ (%)** | **N_2C_/N_3C_** |
| --- | --- | --- | --- |
| CNS | 43.18 | 48.44 | 1.12 |
| S-CNS | 44.48 | 47.38 | 1.06 |
| CNT | 51.6 | 36.74 | 0.71 |
| S-CNT | 30.35 | 60.55 | 2.00 |

**Table S2**. The EXAFS curves fitting parameters.

| **Samples** | **Path** | **N** | **R (Å)** | **σ^2^ (Å^2^)** | **∆E_0_ (eV)** | **R factor** |
| --- | --- | --- | --- | --- | --- | --- |
| S-CNS-Zn | Zn-N | 2.9±0.5 | 1.97±0.02 | 0.012±0.010 | 1.9±1.3 | 0.011 |
|  | Zn-O | 1.0 | 1.96±0.01 | 0.002±0.002 |  |  |
| S-CNT-Zn | Zn-N | 2.6±0.7 | 1.99±0.02 | 0.009±0.002 | -1.67±1.4 | 0.009 |
|  | Zn-O | 1.0 | 1.92±0.01 | 0.018±0.007 |  |  |

Note: S_0_^2^ was fixed as 0.72. N, coordination number; R, distance between absorber and backscatter atoms; σ^2^, Debye–Waller factor to account for both thermal and structural disorders; ΔE_0_, inner potential correction.

**Table S3**. The electronic properties of the *OO intermediate.

| **Property** | **S-CNS** | **S-CNS-Zn** | **S-CNT** | **S-CNT-Zn** |
| --- | --- | --- | --- | --- |
| *OO Bader charge (e^–^) | 0.36 | 0.57 | 0.15 | 0.58 |
| O=O bond length (Å) | 1.28 | 1.47 | 1.25 | 1.33 |

**Table S4**. Summary of some reported carbon-based photocatalysts for H_2_O_2_ production.

| **Catalyst** | **Production rate (μmol g^–1^ h^–1^)** | | **AQY** | **SCC** | **Irradiation conditions** | **Reaction solution, sample weight** | **Ref.** |
| --- | --- | --- | --- | --- | --- | --- | --- |
| S-CNT-Zn | 2708 | 9.88% at 420 nm | | 0.52% | 300 W Xe lamp (λ > 400 nm) | 50 mL 20 vol% EtOH, 2.5 mg | This work |
| S-CNS-Zn | 1724 | 6.28% at 420 nm | | 0.37% | 300 W Xe lamp (λ > 400 nm) | 50 mL 20 vol% EtOH, 2.5 mg | This work |
| Defective C_3_N_4_ | 1620 | 4.6% at 420 nm | | N | Solar simulator (λ > 400 nm) | 60 mL 20 vol% IPA, 50 mg | [S11] |
| g-C_3_N_4_-CNTs | 130 | N | | N | 300 W Xe lamp (λ > 400 nm) | 100 mL 5 vol% FA, 100 mg | [S12] |
| fl-CN | 1470 | 9.0 % at 420 nm | | N | 300 W Xe lamp (λ > 420 nm) | 50 mL 10 vol% EtOH, 25 mg | [S13] |
| NMT_400_ | 1700 | 2.6% at 420 nm | | N | AM1.5G | 50 mL 10 vol% EtOH, 20 mg | [S14] |
| *ds*-BCN | 2256 | 7.6% at 400 nm | | 0.3% | 300 W Xe lamp (λ > 400 nm) | 50 mL 4 vol% BA, 10 mg | [S15] |
| S-CN | 566.69 | 2.12% at 420 nm | | N | 300 W Xe lamp (λ > 420 nm) | 100 mL 10 vol% EtOH, 50 mg | [S16] |
| K, P, O-CN | 485.7 | 4.0% at 420 nm | | N | 300 W Xe lamp (λ > 420 nm) | 40 mL 10 vol% EtOH, 20 mg | [S17] |
| SCBCN_0.4_ | 620 | N | | N | 300 W Xe lamp (λ > 420 nm) | 100 mL 10 vol% EtOH, 40 mg | [S18] |
| B-CN | 574 | 5.7% at 450 nm | | N | 300 W Xe lamp (λ > 420 nm) | 100 mL 10 vol% IPA, 50 mg | [S19] |
| CN/WO | 730 | N | | N | 300 W Xe lamp (λ > 420 nm) | 25 mL 10 vol% IPA, 50 mg | [S20] |
| Nv-C_3_N_4_ | 288.89 | 1.6% at 400 nm | | N | λ > 400 nm | 100 mL 10 vol% IPA, 50 mg | [S21] |
| MCN/CdS | 710 | N | | N | 300 W Xe lamp (λ > 420 nm) | 100 mL 10 vol% EtOH, 40 mg | [S22] |
| ACN | 1847 | 4.4% at 400 nm | | N | 300 W Xe lamp (λ > 420 nm) | 50 mL 10 vol% EtOH, 25 mg | [S23] |
| PEI/CN | 208.1 | 2.21% at 420 nm | | 0.05% | Xe-lamp AM 1.5G | 20 mL water, 20 mg | [S24] |
| BCN-OH_2/_Co_1_ | 60 | 8.0% at 420 nm | | 0.6% | LED lamp | 30 mL water, 20 mg | [S25] |
| NMP | 1965 | 8.4% at 420 nm | | N | AM 1.5G | 10 mL 10 vol% EtOH, 50 mg | [S26] |
| CN/MTI | 64.5 | 5.1% at 420 nm | | 0.18% | 300 W Xe lamp (λ > 420 nm) | 30 mL water, 50 mg | [S27] |
| DMCR-1NH | 2580 | 8.8% at 420 nm | | N | 300 W Xe lamp (λ > 420 nm) | 20 mL 10 vol% EtOH, 10 mg | [S28] |
| CN/BDI_50_ | 52.4 | 1.57% at 420 nm | | 0.13% | Visible light (λ > 420 nm) | 15 mL water, 10 mg | [S29] |
| CNIO-GaSA | 6.6 | 7.1% at 459 | | 0.4% | Visible light (λ > 420 nm) | 2 mL H_2_O, 2 mg | [S30] |
| P-CN | 211 | 1.8% at 420 nm | | 0.14% | 300 W Xe lamp (λ > 420 nm) | 20 mL water, 20 mg | [S31] |
| Co_1_/AQ/C_3_N_4_ | 124 | N | | N | AM 1.5G | 50 mL water, 25 mg | [S32] |
| Sb-SAPC15 | 588 | 17.6% at 420 nm | | 0.61% | Visible light (λ > 420 nm) | 50 mL water, 100 mg | [S33] |
| SA-TCPP | 80 | N | | N | Visible light (λ > 420 nm) | 50 mL water, 25 mg | [S34] |

**Supplementary References**

[S1] J. P. Perdew, K. Burke and M. Ernzerhof, *Phys. Rev. Lett.*, **1997**, 78, 1396-1396.

[S2] R. P. M. Moreira and G. Li Puma, *Catal. Today*, **2021**, 361, 77-84.

[S3] G. Kresse and D. Joubert, Phys. *Rev. B.*, 1**999**, 59, 1758-1775.

[S4] G. Kresse and J. Furthmuller, *Phys. Rev. B.*, **1996**, 54, 11169-11186.

[S5] H. J. Monkhorst and J. D. Pack, *Phys. Rev. B.*, **1976**, 13, 5188-5192.

[S6] W. Tang, E. Sanville and G. Henkelman, *J. Phys.-Condens. Matter*, **2009**, 21, 084204.

[S7] E. Sanville, S. D. Kenny, R. Smith and G. Henkelman, *J. Comput. Chem.*, **2007**, 28, 899-908.

[S8] G. Henkelman, A. Arnaldsson and H. Jonsson, *Comp. Mater. Sci.*, **2006**, 36, 354-360.

[S9] M. Yu and D. R. Trinkle, *J. Chem. Phys.*, **2011**, 134, 064111.

[S10] V. Wang, N. Xu, J.-C. Liu, G. Tang and W.-T. Geng, *Comput. Phys. Commun.*, **2021**, 267, 108033.

[S11] L. Shi, L. Yang, W. Zhou, Y. Liu, L. Yin, X. Hai, H. Song and J. Ye, *Small*, **2018**, 14, 1703142.

[S12] M. Baca, M. Dworczak, M. Aleksandrzak, E. Mijowska, R. J. Kalenczuk and B. Zielinska, *Int. J. Hydrogen Energy.*, **2020**, 45, 8618-8628.

[S13] B. Feng, Y. Liu, K. Wan, S. Zu, Y. Pei, X. Zhang, M. Qiao, H. Li and B. Zong, *Angew. Chem. Int. Ed.*, **2024**, 63, e202401884.

[S14] C. Yang, S. Wan, B. Zhu, J. Yu and S. Cao, *Angew. Chem. Int. Ed.*, **2022**, 61, e202208438.

[S15] F. Chen, X. Lv, H. Wang, F. Wen, L. Qu, G. Zheng and Q. Han, *Jacs Au*, **2024**, 4, 1219-1228.

[S16] C. Feng, L. Tang, Y. Deng, J. Wang, Y. Liu, X. Ouyang, H. Yang, J. Yu and J. Wang, *Appl. Catal. B-Environ.*, **2021**, 281, 119539.

[S17] G.-h. Moon, M. Fujitsuka, S. Kim, T. Majima, X. Wang and W. Choi, *Acs Catal.*, **2017**, 7, 2886-2895.

[S18] Q. You, C. Zhang, M. Cao, B. Wang, J. Huang, Y. Wang, S. Deng and G. Yu, *Appl. Catal. B-Environ.*, **2023**, 321, 121941.

[S19] C. Feng, L. Tang, Y. Deng, J. Wang, J. Luo, Y. Liu, X. Ouyang, H. Yang, J. Yu and J. Wang, *Adv. Funct. Mater.*, **2020**, 30, 2001922.

[S20] T. Shao, Y. Chang, Z. Li, Y. Song, D. Jin, J. Gao, L. Sun and J. Hou, *J. Mater. Chem. A.*, **2023**, 11, 1199-1207.

[S21] X. Qu, S. Hu, P. Li, Z. Li, H. Wang, H. Ma and W. Li, *Diamond Relat. Mater.*, **2018**, 86, 159-166.

[S22] S. Jing, J. Zhao, A. Wang, Q. Ji, R. Cheng, H. Liang, F. Chen, P. Kannan, A. Brouzgou and P. Tsiakaras, *Chem. Eng. J.*, **2024**, 479, 147150.

[S23] Y. Zheng, Y. Luo, Q. Ruan, S. Wang, J. Yu, X. Guo, W. Zhang, H. Xie, Z. Zhang and Y. Huang, *Appl. Catal. B-Environ.*, **2022**, 311, 121372.

[S24] X. Zeng, Y. Liu, Y. Kang, Q. Li, Y. Xia, Y. Zhu, H. Hou, M. H. Uddin, T. R. Gengenbach, D. Xia, C. Sun, D. T. McCarthy, A. Deletic, J. Yu and X. Zhang, *Acs Catal.*, **2020**, 10, 3697-3706.

[S25] H. Ou, Y. Jin, B. Chong, J. Bao, S. Kou, H. Li, Y. Li, X. Yan, B. Lin and G. Yang, *Adv. Mater.*, **2024**, 36, 2404851.

[S26] C. Yang, Y. Xiang, W. Wang, B. Cheng, K. Yang, J. Yu and S. Cao, *Appl. Catal. B-Environ.*, **2025**, 365, 124856.

[S27] Y. Kofuji, S. Ohkita, Y. Shiraishi, H. Sakamoto, S. Ichikawa, S. Tanaka and T. Hirai, *Acs Sustain. Chem. Eng.*, **2017**, 5, 6478-6485.

[S28] P. Das, G. Chakraborty, J. Roeser, S. Vogl, J. Rabeah and A. Thomas, *J. Am. Chem. Soc.*, **2023**, 145, 2975-2984.

[S29] J. Cao, H. Wang, Y. Zhao, Y. Liu, Q. Wu, H. Huang, M. Shao, Y. Liu and Z. Kang, *J. Mater. Chem. A.*, **2020**, 8, 3701-3707.

[S30] H. Tan, P. Zhou, M. Liu, Q. Zhang, F. Liu, H. Guo, Y. Zhou, Y. Chen, L. Zeng, L. Gu, Z. Zheng, M. Tong and S. Guo, *Nat. Synth.*, **2023**, 2, 557-563.

[S31] X. Zhang, P. Ma, C. Wang, L. Gan, X. Chen, P. Zhang, Y. Wang, H. Li, L. Wang, X. Zhou and K. Zheng, *Energy Environ. Sci.*, **2022**, 15, 830-842.

[S32] C. Chu, Q. Zhu, Z. Pan, S. Gupta, D. Huang, Y. Du, S. Weon, Y. Wu, C. Muhich, E. Stavitski, K. Domen and J. H. Kim, *Proc. Natl. Acad. Sci.*, **2020**, 117, 6376-6382.

[S33] Z. Teng, Q. Zhang, H. Yang, K. Kato, W. Yang, Y.-R. Lu, S. Liu, C. Wang, A. Yamakata, C. Su, B. Liu and T. Ohno, *Nat. Catal.*, **2021**, 4, 374-384.

[S34] Y. Zhang, C. Pan, G. Bian, J. Xu, Y. Dong, Y. Zhang, Y. Lou, W. Liu and Y. Zhu, *Nat. Energy.*, **2023**, 8, 361-371.
